# Deaminase-Independent Inhibition of Parvoviruses by the APOBEC3A Cytidine Deaminase

**DOI:** 10.1371/journal.ppat.1000439

**Published:** 2009-05-22

**Authors:** Iñigo Narvaiza, Daniel C. Linfesty, Benjamin N. Greener, Yoshiyuki Hakata, David J. Pintel, Eric Logue, Nathaniel R. Landau, Matthew D. Weitzman

**Affiliations:** 1 Laboratory of Genetics, The Salk Institute for Biological Studies, La Jolla, California, United States of America; 2 Department of Microbiology, New York University School of Medicine, New York, New York, United States of America; 3 Department of Molecular Microbiology and Immunology, University of Missouri–Columbia, School of Medicine, Life Sciences Center, Columbia, Missouri, United States of America; University of Southern California School of Medicine, United States of America

## Abstract

The APOBEC3 proteins form a multigene family of cytidine deaminases with inhibitory activity against viruses and retrotransposons. In contrast to APOBEC3G (A3G), APOBEC3A (A3A) has no effect on lentiviruses but dramatically inhibits replication of the parvovirus adeno-associated virus (AAV). To study the contribution of deaminase activity to the antiviral activity of A3A, we performed a comprehensive mutational analysis of A3A. By mutation of non-conserved residues, we found that regions outside of the catalytic active site contribute to both deaminase and antiviral activities. Using A3A point mutants and A3A/A3G chimeras, we show that deaminase activity is not required for inhibition of recombinant AAV production. We also found that deaminase-deficient A3A mutants block replication of both wild-type AAV and the autonomous parvovirus minute virus of mice (MVM). In addition, we identify specific residues of A3A that confer activity against AAV when substituted into A3G. In summary, our results demonstrate that deaminase activity is not necessary for the antiviral activity of A3A against parvoviruses.

## Author Summary

The APOBEC3 proteins constitute a family of seven cytidine deaminases. Cytidine deaminases are editing enzymes able to remove the amine group from cytidine in single-strand DNA (ssDNA) and RNA, converting it to uracil. APOBEC3 proteins have potent antiviral activity against retroviruses, retrotransposons, and DNA viruses. APOBEC3 generated high interest because of the ability of APOBEC3G (A3G) to inhibit HIV. APOBEC3A (A3A) is a member of the family that inhibits the human parvovirus adeno-associated virus (AAV) and the retrotransposon LINE-1. Parvoviruses are simple ssDNA viruses that do not require a retrotranscription step for their replication. In contrast to A3G, which is predominantly cytoplasmic, A3A is located in both the nucleus and cytoplasm. In addition, A3A consists of a single cytidine deaminase catalytic domain, whereas A3G has two. The dependence of the antiviral function on deaminase activity is controversial. In this study, we identify numerous A3A residues required for deaminase and antiviral activities. We show that A3A not only inhibits AAV but also the minute virus of mice (MVM). Importantly, we demonstrate that A3A does not require its deaminase activity to block the replication of both parvoviruses. Thus, exploiting the simplicity of parvoviruses together with the single-domain cytidine deaminase A3A, we are able to demonstrate that cytidine deaminase activity is not required for APOBEC3 mediated viral inhibition.

## Introduction

Eukaryotes have evolved numerous innate immune defenses against invading pathogens. The apolipoprotein B mRNA-editing catalytic polypeptide-like 3 (APOBEC3) proteins comprise a family of seven cytidine deaminases [1]–[4] that may each form distinct intrinsic barriers to endogenous retrotransposons and invading viruses [5]–[7]. The most characterized member of the family is APOBEC3G (A3G), which restricts Vif-deficient human immunodeficiency virus 1 (HIV-1) [8]–[11]. In addition to HIV-1, the APOBEC3 proteins inhibit a diverse array of viruses including simian immunodeficiency virus (SIV), human T cell leukemia virus 1 (HTLV1), murine leukemia virus (MLV), mouse mammary tumor virus (MMTV), and hepatitis B virus (HBV) [6],[7],[10]–[13]. Interestingly, human APOBEC3A (A3A) lacks activity against retroviruses but dramatically inhibits replication of the parvovirus adeno-associated virus (AAV) [14]. The molecular mechanisms that govern specificity of APOBEC3 antiviral activity are not yet fully understood.

The APOBEC3 proteins share structural and functional features with zinc-dependent deaminases and possess cytidine deaminase activity [1],[4]. The cytidine deaminase domains (CDDs) of APOBEC3 proteins contain an active site with the conserved consensus motif H-X-E-X_23–28_-P-C-X_2–4_-C (where X is any amino acid). It has been proposed that the histidine and the two cysteine residues coordinate a Zn^2+^ ion, while the glutamic acid residue serves an essential role in catalysis as a proton shuttle [15]–[18]. APOBEC3 proteins contain either a single CDD (A3A, A3C and A3H) or two tandem CDDs (A3B, A3D/E, A3F and A3G). In the case of A3G, both domains contain an intact active site consensus sequence motif but only CDD2 appears to be catalytically active, while CDD1 is responsible for interaction with the HIV nucleocapsid proteins and packaging [19]–[23]. Sequence alignment shows that A3A is highly homologous to the C-terminus of A3B and A3G proteins [2].

The mechanism of the antiviral activity of APOBEC3 proteins against retroviruses has been studied extensively [5],[6],[24]. APOBEC3 proteins are incorporated into virus particles, and encapsidation is mediated via interactions with Gag, viral RNA and cellular RNAs [19],[22],[23],[25]–[32]. Encapsidated A3G is delivered into target cells where it deaminates dC to dU on newly synthesized minus strand cDNAs during the process of reverse transcription [9]–[11],[33]–[35]. Deaminated genomes can be degraded by the action of the cellular base excision repair machinery [36], although recent reports suggest that uracil-DNA glycosylase 2 (UNG2) is not required for the A3G antiviral function [37],[38]. In addition, hypermutated proviruses will contain sequence changes that inactivate the virus by generating alternate splicing, premature translation, and nonfunctional proteins [39].

Whether cytidine deamination is the principal mechanism for the antiviral activity of APOBEC3 proteins remains controversial [39]. APOBEC3 chimeric proteins and catalytically inactive mutants demonstrate that inhibition of HIV-1 can be achieved by A3G that lacks deaminase activity [40]–[42]. A3G can inhibit lentivirus reverse transcriptase (RT) and prevent accumulation of reverse transcripts and viral cDNA in target cells in a deamination-independent manner [35],[42]–[44]. Additionally, subsequent steps of viral integration have been shown to be affected by APOBEC3 proteins [37],[45]. The idea of deaminase-independent antiviral activity has been challenged by others who have argued that cytosine deamination is required for efficient inhibition of retroelements at low levels of APOBEC3 expression [38],[46],[47]. In the case of HBV, the mechanism for inhibition by APOBEC3 proteins is also controversial but has been suggested to be deaminase-independent due to infrequent editing, and may be caused by blocking reverse transcription and expression of HBV antigens [12],[48],[49].

Although it lacks activity against retroviruses, A3A is a potent inhibitor of both parvovirus and the human transposon LINE-1 [14],[50],[51]. Parvoviruses are small eukaryotic viruses that infect humans and a variety of other animal species [52]. The parvovirus genome consists of a linear single-stranded DNA (ssDNA) molecule approximately 4.5 kb in length, with hairpin structures at both ends that function as origins for viral DNA replication. The genome contains two major ORFs that encode the nonstructural replication proteins (NS or Rep) and the structural capsid proteins (Cap). The family is divided into autonomous parvoviruses and dependoviruses, which require a helper virus for efficient replication and progeny production. The minute virus of mice (MVM) is an autonomous parvovirus while adeno-associated virus (AAV) is a dependovirus that uses adenovirus as a helper virus. In our previous studies we found that despite the absence of detectable AAV editing, mutations in conserved active site residues of A3A abrogated the antiviral activity against AAV [14].

A3A inhibition of parvovirus provides an attractive system in which to decipher the relative contribution of deamination and deaminase-independent mechanisms to antiviral activity. Unlike the other viruses known to be inhibited by APOBEC3 proteins, the parvoviruses replicate exclusively in the nucleus, do not pass through RNA intermediates, do not have a reverse transcription step in their replication schemes, and use DNA hairpins for priming replication [52]. Moreover, A3A is a simplified system for probing APOBEC3 functions because it has a single CDD and is not restricted to a specific subcellular compartment [2],[14].

To ascertain whether deaminase activity is required for inhibition of parvovirus replication, and to understand the functional significance of amino acid divergence between A3A and A3G, we analyzed the properties of a panel of A3A point mutants and A3A/A3G chimeras. The proteins were tested for deaminase activity *in vitro* and for antiviral activity in rAAV production assays. We identify mutants that lack deaminase activity but retain the antiviral effect, supporting the idea of a deamination-independent mechanism. In addition to AAV, we show that A3A inhibits DNA replication of the autonomous parvovirus MVM. Chimeric A3A/A3G proteins generated by exchanging divergent sequences demonstrate loss-of-function for A3A and gain-of-function for A3G. Our mutants also reveal residues in the linker and pseudoactive site domains that are important for deamination, target specificity and antiviral activities of A3A. Together, these studies reveal domains of A3A that are responsible for its distinct antiviral activity and suggest that A3A can inhibit parvoviruses through a mechanism separate from its function as a cytidine deaminase.

## Results

### Inhibition of rAAV Production Is Not Dependent Upon the Deaminase Activity of A3A

**Figure 1:**
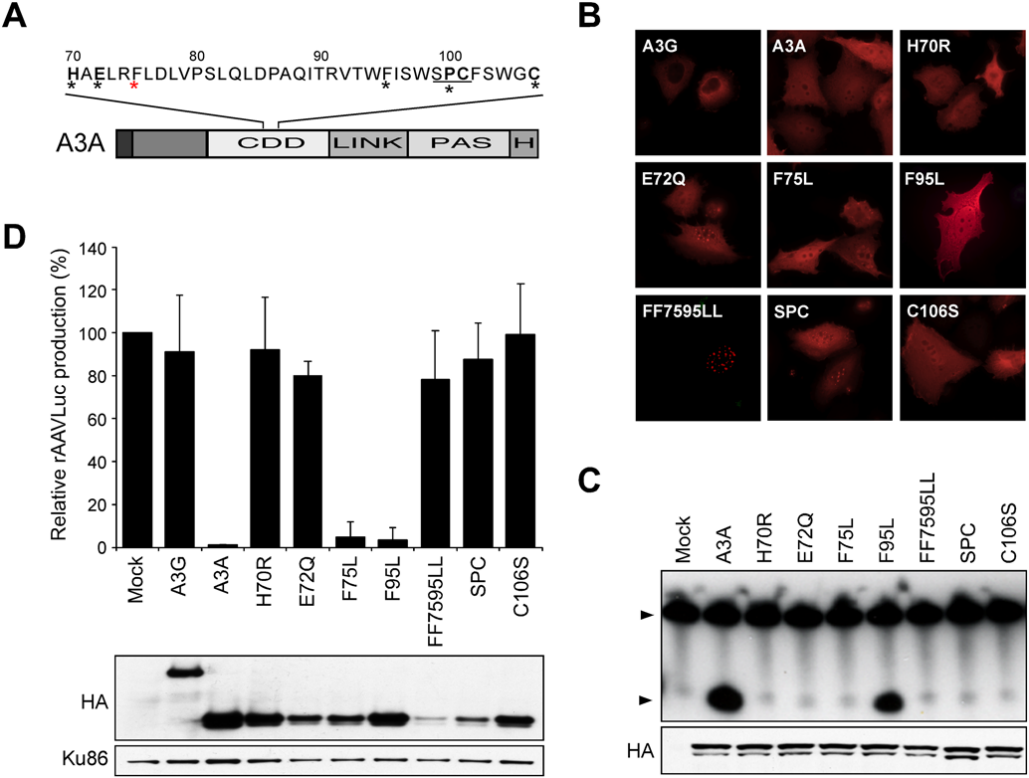
Deamination is not required for antiviral activity of A3A. (A) Schematic of A3A and active site mutants. Domains marked are the cytidine deaminase domain (CDD), the linker (LINK), the pseudoactive site (PAS) and the hemagglutinin epitope tag (H). Active site residues conserved among APOBEC3 proteins (H-X-E-X_28_-PC-X_4_-C) are indicated in bold. Asterisks indicate specific mutations generated for this study; F75 is indicated with a red asterisk. (B) Immunofluorescence to detect HA-tagged APOBEC3 proteins (red) expressed by transfection in U2OS cells. (C) *In vitro* assay for cytidine deaminase activity. Proteins were generated by IVT, immuno-precipitated by the HA epitope, and incubated with a radiolabeled substrate (T_28_TCAT_29_). The deaminated molecules were cleaved by treatment with UDG followed by high pH, and the products were resolved by PAGE (upper panel). Arrows indicate the substrate and deaminated product. The panel below shows an immunoblot to detect *in vitro* translated proteins. (D) Inhibition of AAV. Production of rAAVLuc was assessed by transfection of 293T cells with AAV plasmids in the presence of APOBEC3 expression constructs (1 µg). Production of rAAV was assessed by transduction of target cells and quantitation of luciferase activity. Presented is the average of three independent experiments normalized to vector only control (mock). The panels below show immunoblots to detect HA-tagged wild type and mutant A3A proteins in transfected 293T cell lysates. Ku86 served as a loading control.

We previously showed that A3A antiviral activity was dependent on the integrity of conserved amino acids in the active site that are responsible for proton shuttling and zinc coordination [14]. These results suggested that the catalytic domain of A3A must be intact for antiviral activity against AAV. To extend these studies, we generated additional point mutations at conserved residues (F75 and F95) previously shown to be required for deaminase activity of APOBEC1 [16],[53]. We also mutated the 99SPC101 residues to AAA (SPC) in the active site domain of A3A [50]. The position of these mutants is indicated in Figure 1A.

First, we determined the cellular localization of the mutant proteins in transfected U2OS and HeLa cells (Figure 1B and data not shown). Wild-type A3A was located throughout the cell, while A3G was predominantly cytoplasmic (Figure 1B). Most of the A3A mutants displayed cellular localization patterns similar to wild-type protein. The double mutant FF7595LL showed a nuclear punctate pattern (also observed in some cells transfected with E72Q and SPC), which might reflect misfolded protein.

We next measured the catalytic activity of the proteins using an *in vitro* deamination assay. We previously demonstrated deaminase activity for A3A packaged into HIV-1 virions [14]. To bypass the requirement for packaging, we directly immunoprecipitated A3A from lysates of transfected cells [47]. We further adapted the assay to control for different expression levels by generating A3A by coupled *in vitro* transcription/translation (IVT) using wheat germ extract. An advantage of this method is that it allows assessment of proteins that are unstable or difficult to express in cells, such as FF7595LL and A3A truncations (Figure S1). Cytidine deaminase activity of the immunoprecipitates was measured by incubation with a radiolabeled deoxyoligonucleotide containing a single deoxycytidine target site. Wild-type A3A protein generated by IVT had deaminase activity (Figure 1C) and demonstrated similar activity to A3A produced by transfection (Figure S1). These results showed that the IVT generated protein was catalytically active and also suggested that A3A does not require a mammalian cellular co-factor for its catalytic activity. Analysis of the active site mutants in the IVT system showed that, consistent with previous studies, mutants in conserved active site amino acids (H70R, E72Q, C106S, and SPC) had lost deaminase activity [14],[50]. Although the F75 and F95 residues have both been shown to be required for APOBEC1 deaminase activity, the F95L mutant of A3A retained deaminase activity in our analysis, while the F75L mutant was inactive.

The antiviral activity of A3A mutants was compared to wild-type A3A and A3G by transfection of 293T cells in the recombinant AAV (rAAV) production assay (Figure 1D). The APOBEC3 expression vectors were cotransfected with plasmids required for rAAV replication and packaging. The Rep and Cap proteins were supplied in *trans* to allow replication of an AAV vector and packaging of the ssDNA genome into virus particles. In this study we employed rAAV expressing luciferase (rAAVLuc), which allowed for quantitative assessment of virus production by transduction of target cells. Immunoblotting confirmed that proteins of the expected size were expressed (Figure 1D). Wild-type A3A completely blocked rAAV production, as previously reported [14]. In contrast, neither A3G nor the active site A3A mutants H70R, E72Q, SPC and C106S inhibited rAAV production. The F95L mutant that retains deaminase activity was active against rAAV. Surprisingly, F75L also inhibited rAAV despite its lack of deaminase activity in the *in vitro* assay. The F75L protein is therefore a separation-of-function mutant of A3A that facilitates analysis of the relative contribution of the deaminase-dependent and -independent mechanisms to AAV inhibition. The double mutant FF7595LL did not inhibit rAAV production but was poorly expressed and showed altered cellular distribution. Together, these data demonstrate that deaminase activity is not required for the anti-AAV effect of A3A.

### A3A Inhibits Parvovirus Replication by a Deaminase-Independent Mechanism

**Figure 2:**
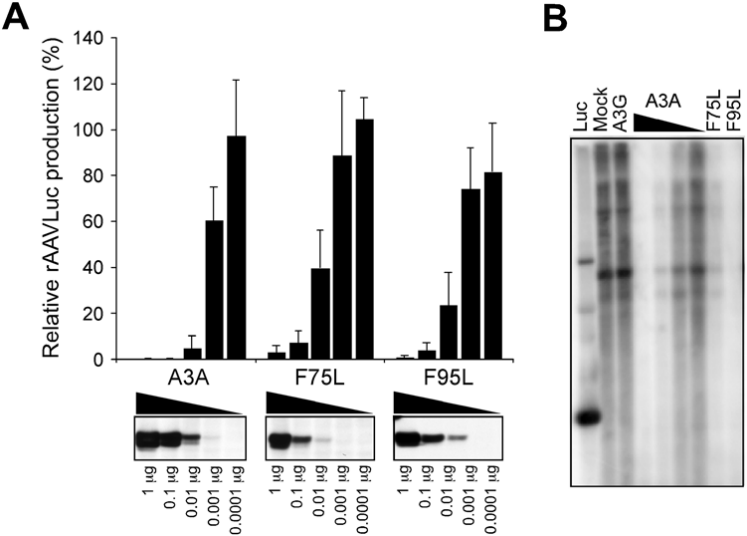
A3A mutants inhibit AAV DNA replication. (A) Titration of wild-type and mutant A3A expression vectors in rAAVLuc production assays. Production of rAAVLuc was assessed by transduction of target cells and quantitation of luciferase activity. Presented is the average of four independent experiments normalized to vector alone control (mock). The panels below show immunoblots to detect HA-tagged wild-type and mutant A3A proteins in transfected 293T cell lysates. (B) Southern blot detection of low molecular weight DNA extracted from 293T cells transfected for rAAVLuc production in the presence of mock (1 µg) A3G (1 µg), A3A (1, 0.1, 0.01 and 0.001 µg) and mutant A3A expression vectors (1 µg). The DNA was digested with *Dpn*-I, separated by gel electrophoresis, and hybridized with a radiolabeled luciferase probe.

To exclude differences in AAV inhibition due to disparities in protein expression levels, we compared the mutants F75L and F95L with wild-type A3A over a dose-response (Figure 2A). Immunoblotting revealed that expression levels of the mutants F75L and F95L were approximately ten-fold lower than wild-type A3A when equal amounts of DNA were transfected. However, despite differences in the deamination ability of the mutants, they displayed similar antiviral activity to wild-type A3A when equivalent protein levels were compared. We tested the F75L mutant against a panel of oligonucleotides that contained a cytosine in each of the possible tri-nucleotide configurations, but found no evidence of deamination above background levels on any sequence (Figure S2A and Table S1). Therefore the lack of detectable deaminase activity for F75L *in vitro* was not caused by an altered target sequence preference. We also tested the deaminase activity of A3A mutants in cell lysates by adapting the quantitative fluorescence resonance energy transfer (FRET) assay recently developed for A3G [54]. This FRET assay measures cleavage of a target oligonucleotide dual-labeled with fluorophores (Figure S2B). We observed dose-dependent deaminase activity with increasing amounts of cell lysates from 293T cells transfected with the A3A plasmid. Background levels of activity were obtained with the defective mutants E72Q and C106S. F95L showed deaminase activity in this assay, whereas F75L was not above background (Figure S2B). This result supports the observations from the *in vitro* deaminase assay and the conclusion that AAV is inhibited in the absence of deamination.

In our previous studies of AAV production in the presence of wild-type A3A, we found no detectable evidence of AAV sequence changes but viral replication was inhibited [14]. To detect effects of A3A on the accumulation of rAAV DNA, we used Southern blotting of low molecular weight DNA extracted from the transfected cells during rAAV production (Figure 2B). As controls, we compared the A3A mutants to wild-type A3A and A3G. Replicated rAAV DNA was detected by hybridization with a luciferase probe. Although F75L was slightly less effective than F95L, both mutants inhibited the accumulation of rAAV DNA (Figure 2B), suggesting that inhibition of AAV replication is not dependent on deamination.

**Figure 3:**
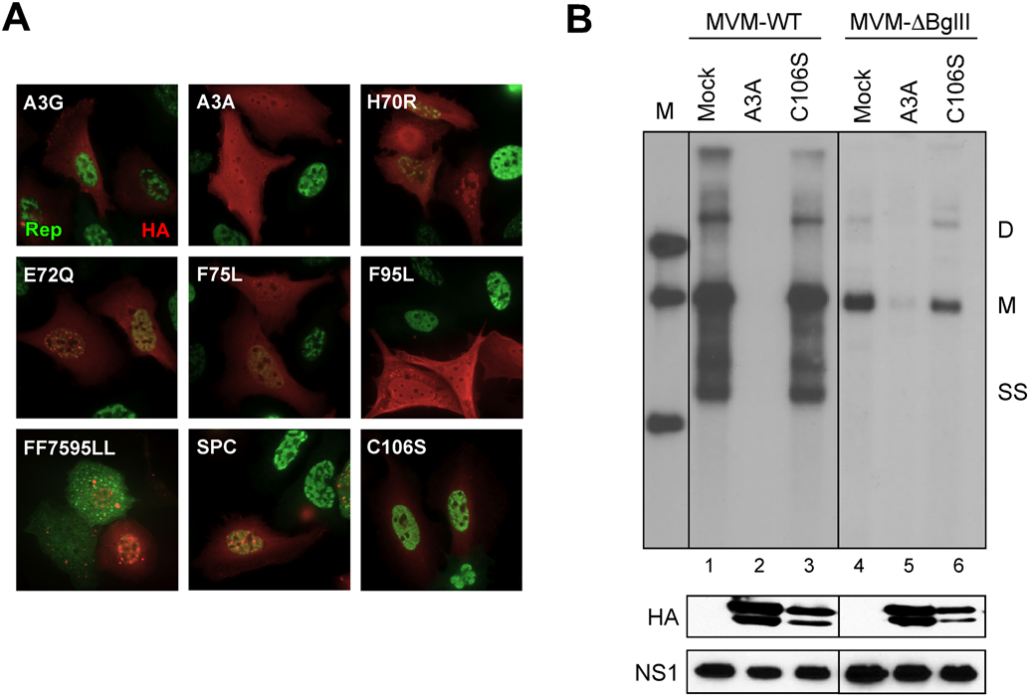
A3A inhibits AAV2 and MVM DNA replication. (A) Inhibition of wild-type AAV replication. U2OS cells were transfected with plasmids for APOBEC3 proteins and then infected with AAV and adenovirus. HA-tagged APOBEC3 (red) and AAV Rep proteins (green) were detected by immunofluorescence using specific antibodies. (B) Inhibition of wild-type MVM replication. Southern blot detection of low molecular weight DNA extracted from A9 cells cotransfected with an infectious MVM clone together with APOBEC3A expression plasmids. The DNA was digested with *Dpn*-I, separated by gel electrophoresis and hybridized with a radiolabeled MVM probe. Left line is a marker (M). Replicative intermediates of ssDNA (SS), monomer (M), and dimer (D) are indicated to the right. Panel below shows immunoblots for APOBEC3 and the NS1 protein of MVM.

To examine the effect of A3A and mutants on replication of wild-type parvovirus genomes, we used two different viral systems. AAV2 depends upon helper virus for replication, while MVM replicates autonomously. First we used immunofluorescence to assess the effect of APOBEC3 proteins in cells infected with AAV2 and adenovirus helper virus. As previously shown [14], replication centers detected by staining for the viral Rep protein were present in cells that expressed A3G but were absent in those with A3A (Figure 3A). Advanced stage viral replication centers were detected in cells expressing inactive mutants. In contrast, A3A mutants that inhibited rAAV production (F75L and F95L) also blocked formation of viral replication centers. These data demonstrate that observations made with rAAV production also apply to inhibition of wild-type AAV replication.

Replication of an infectious plasmid clone of MVM was also dramatically inhibited by co-transfection of wild-type A3A, but not the C106S mutant (Figure 3B, lanes 1–3). Interestingly, replication of an MVM genome bearing a large in-frame deletion within the capsid gene, and therefore unable to generate the wild-type capsid proteins necessary to produce single-stranded progeny DNA (MVM-Δ*Bgl*II), was also inhibited by A3A (Figure 3B, lanes 4–6). In separate experiments, the expression of the full spectrum of MVM RNA and protein generated from a non-replicating full-length MVM plasmid was not affected by expression of A3A (data not shown), suggesting that A3A directly affects parvovirus genome replication.

### The Linker and Pseudoactive Domains of A3A Are Required for Antiviral Activity

**Figure 4:**
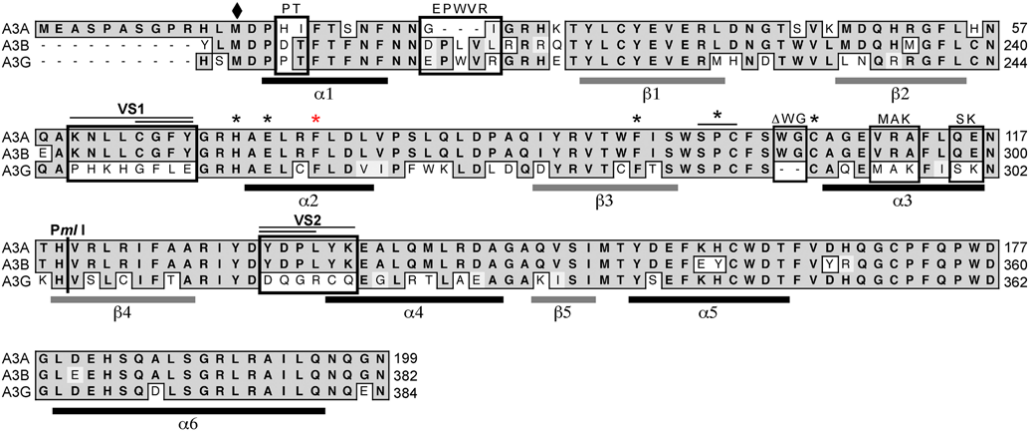
Alignment of APOBEC3 amino acid sequences for A3A with the C-terminus of A3B and A3G. Residues exchanged in A3A are boxed and the mutant designation is indicated above. The *Pml*I site and stretches of variable sequence VS1 and VS2 switched in the chimeras are also indicated. The asterisks mark individual amino acid mutants, and the diamond indicates the start of A3G-CT (residues 197–384). Residue numbers are indicated on the right side. Predicted secondary structure of A3A is indicated below the alignment with α-helices in black and stranded β-sheets in grey. A3A secondary structure modeling was generated by Swiss-Model using the crystal structure of the C-terminal fragment of A3G (Protein Data Bank accession number 3E1A) as template [77].

**Figure 5:**
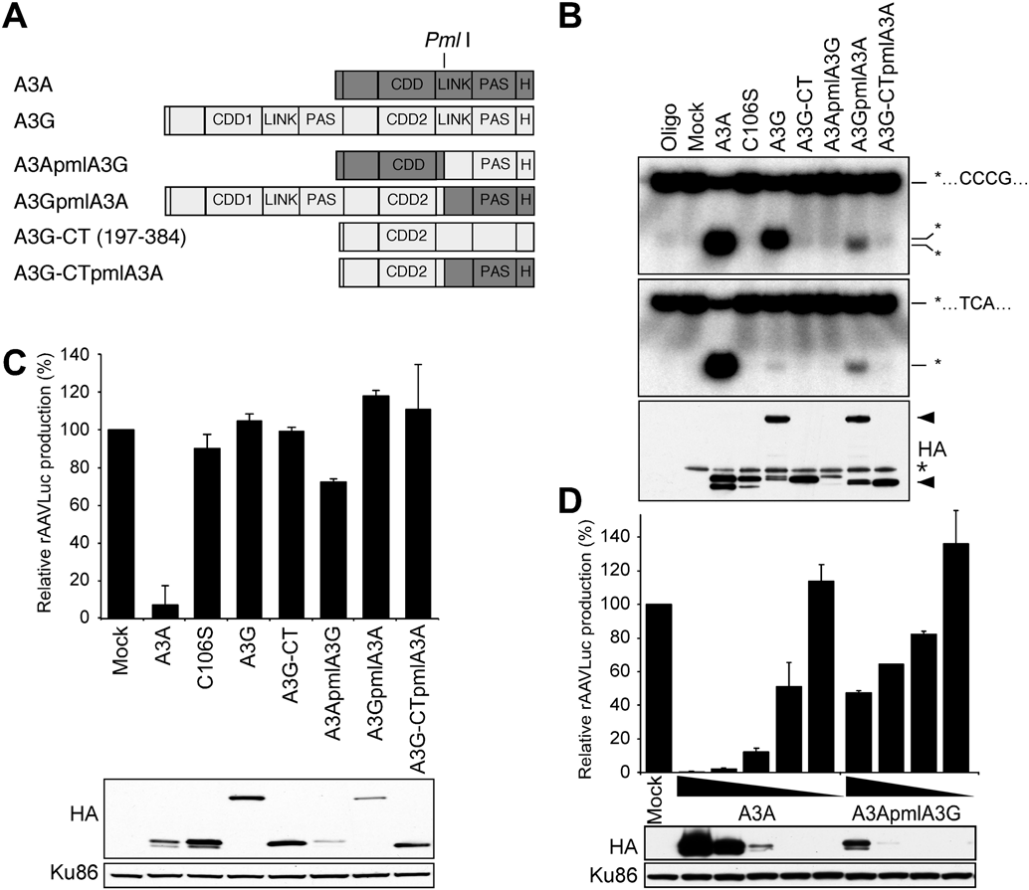
Activity of A3A/A3G P*ml*I based chimeras. (A) Schematic of A3A (dark grey) and A3G (light grey). Domains marked are the cytidine deaminase domains (CDD), the linker (LINK), the pseudoactive site (PAS), and the hemagglutinin epitope tag (H). Below are the chimeras generated at the *Pml*I site. (B) *In vitro* assays for cytidine deaminase activity. Proteins were immunoprecipitated from transfected cells by the HA epitope and incubated with the indicated radiolabeled substrate in UDG-dependent assays. The upper panel uses a substrate oligonucleotide with target sequence CCCG, and the middle panel uses an oligonucleotide with the specific A3A target sequence TCA. The substrate and deaminated products are indicated. The bottom panel shows an immunoblot to detect proteins in immunoprecipitates. A band corresponding to the light-chain IgG used for immunoprecipitation is indicated (*). (C) Production of rAAV in the presence of APOBEC3 proteins. 293T cells were transfected with APOBEC3 constructs (1 µg, except for A3A 0.1 µg), together with plasmids required for rAAVLuc production. Virus production was assessed by transduction of target cells and quantitation by luciferase assay. Panels below show immunoblots for APOBEC3 proteins (HA) in transfected cells and Ku86 as a loading control. (D) Dose-response for A3A and the A3ApmlA3G chimera in the rAAV production assay. Panels below show immunoblots for APOBEC3 (HA) and Ku86 proteins in transfected cells.

To identify further residues required for A3A antiviral activity, we compared the sequence of A3A to the C-terminus of A3B and A3G. Alignment of the amino acid sequences showed that A3A is most closely related to A3B. Two main regions with variable sequences (VS1 and VS2) differ from A3G (Figure 4). Using the structure of the C-terminal domain of A3G as a template [55], we predicted the secondary structure of A3A (Figure 4) and this suggested that the VS1 would be located in the loop between the β2 strand and the α1 helix, and that VS2 partially overlaps with the α4 helix. To define regions of A3A responsible for the antiviral activity against parvoviruses, we tested the contribution of the linker and pseudoactive site subdomains [2]. We first generated chimeric proteins between A3A and A3G (Figure 5A) joined at the shared *Pml*I site. The N-terminus of A3A (residues 1–119) was fused to the C-terminal *Pml*I fragment of A3G (residues 306–384) to form the chimera A3ApmlA3G. The reciprocal chimera A3GpmlA3A was also generated. We also assessed the activity of the C-terminus of A3G (A3G-CT, residues M197 to N384) and a fusion with A3A at the *Pml*I site (A3G-CTpmlA3A). Cellular localization of the mutants was tested by immunofluorescence in transfected cells (Figure S3A). We found that localization of the chimeric proteins to the cytoplasm was determined by the N-terminal domain of A3G as previously reported [51]. We also measured the deaminase activity of chimeric proteins immunoprecipitated from transfected cells in the *in vitro* assay with the T_28_CCCGT_28_ deoxyoligonucleotide substrate (Figure 5B, upper panel). Full-length A3A and A3G both produced robust deamination, but all of the chimeras were less active. The mutants were also tested for activity against rAAV in the transfection assay (Figure 5C). Neither A3G nor A3G-CT had any inhibitory effect against rAAV despite being expressed well in transfected cells. Fusion of the linker and pseudoactive site subdomains of A3A onto A3G or A3G-CT was not sufficient to confer antiviral activity (A3GpmlA3A and A3G-CTpmlA3A). The A3ApmlA3G chimera, which possesses the linker and pseudoactive site of A3G fused onto A3A, showed diminished antiviral activity. This reduction was confirmed in a dose-response titration (Figure 5D). Together these data suggest that the linker and pseudoactive site regions of A3A are important for deamination and antiviral activity, but that these domains are not sufficient to confer activity to A3G.

Careful inspection of the deaminase assay autoradiograms (Figure 5B, upper panel) revealed that the deaminated products for A3A and A3G have slightly different mobilities, which likely reflects differences in deamination target specificity [14],[33],[56]–[59]. The deamination product for A3GpmlA3A migrated similarly to that of A3A, suggesting it had gained the target site specificity of A3A. To test this possibility, we analyzed the deaminase activity on a deoxyoligonucleotide containing the specific A3A target sequence TCA (Table S1). While A3A was highly active on the TCA substrate, A3G was inactive (Figure 5B, middle panel). Although the level of deamination by the A3GpmlA3A chimera was less than that of A3A, this mutant was similarly active on both substrates (Figure 5B, compare upper and middle panels). These observations suggest that the region encompassing the linker and pseudoactive site is involved in target site selection [56]. In our assay the C-terminal fragment of A3G was inactive, although recent studies reported that a similar fragment of A3G (residues 198–384) showed mutator activity in bacteria [17],[60] and deaminase activity *in vitro* [55]. However, these studies used GST-tagged A3G-CT in the bacterial assays or purified recombinant protein in their *in vitro* assays, while we have analyzed protein from cell lysates. Furthermore, A3G-CT was shown to be significantly less active than the full-length protein [55]. Our results may also reflect differences in experimental conditions or that protein produced by transfection may be less active due to RNA inhibition or lack of dimerization.

### Deaminase Activity Is Dispensable for Activity against rAAV

**Figure 6:**
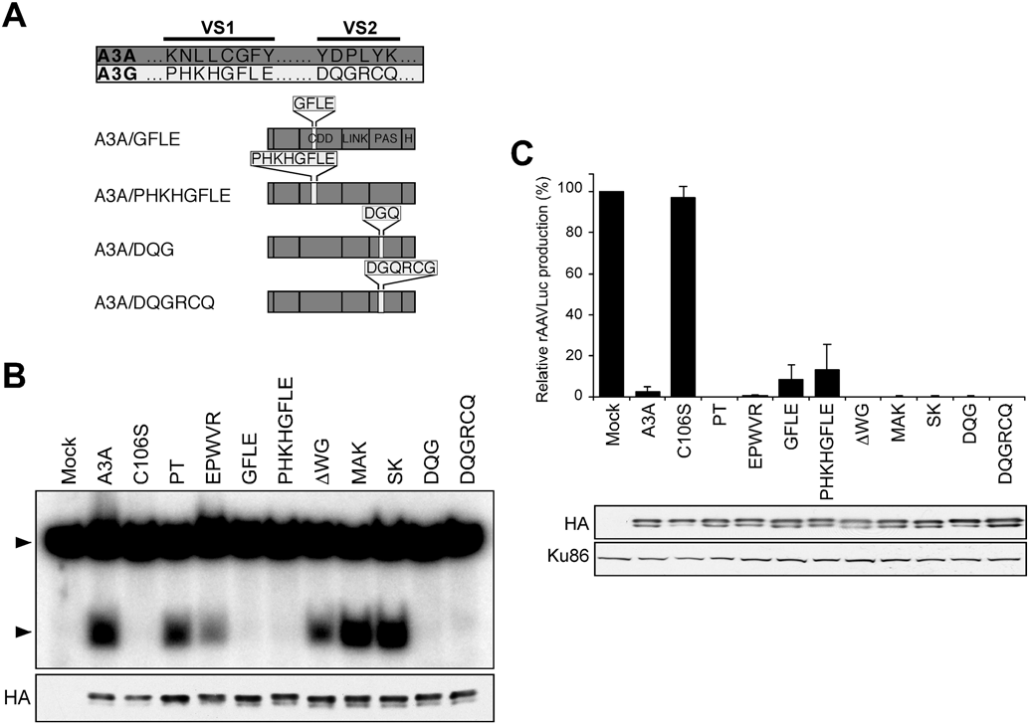
Mutants of A3A with residues replaced with the corresponding sequences of A3G. (A) Schematic of variable segments VS1 and VS2, and the chimeric mutants generated for A3A in these regions. The VS1 segment corresponds to A3A residues 60 to 67, and VS2 corresponds to A3A residues 132 to 137. (B) *In vitro* deamination assay. Proteins were immunoprecipitated from transfected cells and incubated with radiolabeled oligonucleotide (T_28_CCCGT_28_) for 16 h in UDG-dependent assays. Arrows indicate the substrate and deaminated product. The panel below shows an immunoblot to detect proteins in immunoprecipitates. (C) Production of rAAV in the presence of wild-type (0.1 µg) and mutant A3A proteins (1 µg). Panels below show immunoblots for APOBEC3 proteins (HA) in transfected cells and Ku86 as a loading control.

Based on the analysis of chimeric proteins, we generated further mutants in which non-conserved residues of A3A were substituted with those from A3G (see Figure 4). Amino acids that differed between the two proteins were changed throughout A3A (Figure 6A). Most of the mutants consisted of A3A residues replaced with the analogous A3G sequence. Two residues lacking in A3G were deleted from A3A (ΔWG), and in another mutant unique residues from A3G were inserted into A3A (EPWVR). The two main variable regions that contain stretches of divergence (VS1 and VS2 in Figure 4) were switched in two stages that changed 3 or 4 residues at a time (Figure 6A). All mutants displayed the same pattern of cellular localization as wild-type A3A (Figure S3B). Mutant proteins were synthesized by IVT and evaluated for deaminase activity in the *in vitro* assay (Figure 6B). Mutants PT, MAK, and SK retained wild-type levels of activity. The EPWVR and ΔWG mutants had diminished activity compared to wild-type A3A. The chimeras in variable stretches VS1 and VS2 lacked detectable deaminase activity. When these mutants were included in the rAAV production assay, all of them retained the ability to inhibit AAV (Figure 6C). The VS1 mutants with diminished antiviral activity (GFLE and PHKHGFLE) were also compared to wild-type A3A over a dose response (Figure S4). When similar levels of protein were compared for their effect on rAAV production, the VS1 mutants GFLE and PHKHGFLE showed less activity than wild-type A3A (∼75% inhibition compared to ∼95%) (Figure S4). This observation suggests that the VS1 region in A3A contributes to the antiviral activity. Together these data demonstrate that residues outside of the putative enzymatic active site of A3A, in both the N-terminus and C-terminus, are required for efficient deamination but that this does not correlate with antiviral activity against parvovirus replication.

### Activity against AAV Can Be Conferred to A3G

**Figure 7:**
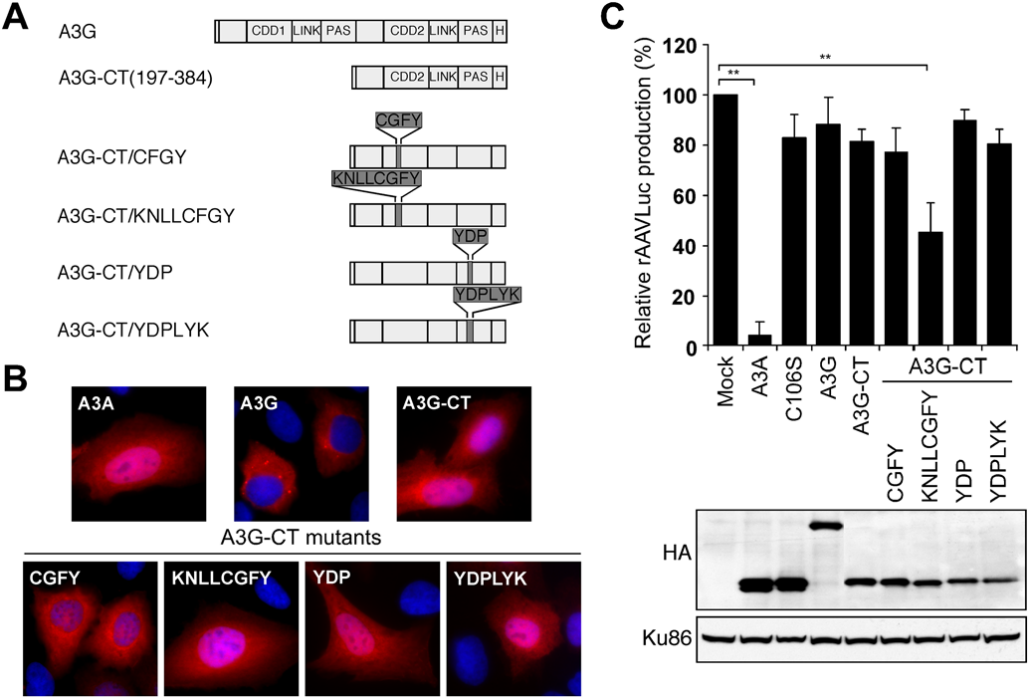
Identification of a gain-of-function mutant for A3G. (A) Schematic of full-length A3G and the C-terminal fragment A3G-CT. Chimeras of A3G-CT were generated with variable segments VS1 and VS2 replaced with sequences of A3A. (B) Immunofluorescence to detect localization of HA-tagged APOBEC3 and chimeric proteins (red) expressed by transfection in U2OS cells. Cell nuclei were detected by staining with DAPI (blue). (C) Production of rAAV in the presence of APOBEC3 proteins. Virus production was assessed by transduction of target cells and quantitation by luciferase assay. Immunoblots show similar expression levels for APOBEC proteins in transfected cells. Ku86 served as a loading control. The A3G-CT/KNLLCGFY mutant demonstrated activity against AAV. The asterisks indicate that the inhibition with A3A and A3G-CT/KNLLCGFY was statistically significant (p<0.001) when compared to mock by Student *t* test.

Analysis of the A3A mutants suggested that the two stretches of residues divergent between A3A and A3G (VS1 and VS2) are important for deamination, and may play a role in antiviral function. Therefore, we determined whether the reciprocal switch (where residues in A3G were substituted with the sequences from A3A) would generate a gain-of-function (Figure 7A). Sequences from A3A were incorporated into constructs that express the C-terminal fragment of A3G which can localize in the nucleus (Figure 7B). The mutant proteins were tested for their effect on AAV in the virus production assay (Figure 7C). Proteins of the expected size were expressed at similar levels. The C-terminal fragment that contained the complete sequence from VS1 of A3A (A3G-CT/KNLLCGFY) acquired significant inhibitory activity against AAV. It was less active than wild-type A3A and reduced rAAV production by approximately 50% when equal levels of protein where compared (Figure S5). When incorporated into full-length A3G, the VS1 region of A3A increased deamination *in vitro*, whereas the VS2 sequences decreased activity (Figure S6). Incorporation of the A3A sequences into full-length A3G did not confer AAV inhibition, presumably due to cytoplasmic localization or interference by the N-terminus (Figure S6). Thus A3G-CT/KNLLCGFY provides the first gain-of-function mutant for A3G and demonstrates that the VS1 region of A3A (residues 60 to 67) contributes to the antiviral activity against parvovirus.

## Discussion

In this report we provide multiple pieces of evidence to show that A3A inhibition of AAV can occur through a deaminase-independent mechanism. Mutation of A3A active site residues that are essential for catalytic activity (H70R, E72Q, SPC99-101AAA and C106S) led to the loss of activity against AAV. However, other mutants (F75L and mutants in VS1 and VS2) separated the deaminase activity from the ability to inhibit AAV. Together these data indicate that the integrity of the active site is important but that deaminase activity is not required for AAV inhibition. In APOBEC1, aromatic residues analogous to F75L and F95L of A3A are required for both deaminase activity and binding to nucleic acids [16],[53]. In the case of A3A, we found that F95 is not required for deaminase activity. This result probably reflects the differences in structure and nucleic acid specificity between APOBEC3 proteins and APOBEC1, as revealed by recent structural studies [17],[55]. We found that the F75L mutant was deaminase-defective in our *in vitro* deaminase assay. The lack of deaminase activity on a panel of target oligonucleotides demonstrated that the F75L A3A mutant is truly deaminase-deficient, and has not simply changed its target site preference and eluded detection in the deaminase assay. Although the *in vitro* deaminase assay has limitations, we demonstrated that lysates containing F75L also lacked deaminase activity in the FRET assay [54]. Evidence that deaminase activity is not essential for inhibition of AAV by A3A is consistent with the absence of signs of deamination in AAV DNA in cells expressing A3A [14].

Deaminase-independent inhibition of ΔVif-HIV and retroelements by A3G has been controversial [35],[39]. The deaminase-defective A3G mutants in E259 retains anti-viral activity when over-expressed [41],[42]. However, when equivalent proteins levels are compared in transient transfections or in stable cell lines, the deaminase deficient mutant has significantly less potent antiviral activity than wild-type A3G [38],[46],[61]. In our studies we assessed the dose response of A3A mutants by comparing their antiviral activity against AAV in titration experiments. We identified deaminase-defective mutants (F75L and mutants in VS2) that displayed similar activity against AAV as wild-type A3A when analyzed at comparable protein levels. In addition, the F75L and VS2 mutants displayed the same subcellular localization as the wild-type protein and thus their phenotype cannot be ascribed to protein mislocalization.

In addition to providing evidence for deaminase-independent antiviral activity, our study also offers insights into the structural basis of APOBEC3 protein function. The linker and pseudoactive site domains in the N-terminus of A3G are required for HIV-1 virion incorporation [19],[62]. We demonstrate that these domains also influence the target site specificity of APOBEC3. A3G prefers the target site (T/C)CC [9],[21],[33],[57],[59], while A3A is more flexible, showing preference for (T/C)CA [14]. Replacement of the linker and pseudoactive sub-domains at the end of A3G with those from A3A modified the target site preference towards the A3A-specific consensus target TCA. Thus, A3A residues in the C-terminus contribute to its target specificity, in agreement with data from chimeric and mutant proteins of other APOBEC3 family members [56]. Residues in the VS2 region have been implicated in target site specificity [1], but in our hands the exchange of VS2 residues between A3A and A3G did not affect target specificity.

It is unclear why A3A has more potent *in vitro* deaminase activity than other APOBEC3 proteins. Deaminase activity of A3G resides in the C-terminal CDD [20],[21],[28],[41],[60], which shares 68% identity with A3A. An intriguing difference between A3A and A3G is the presence of two additional residues (WG) within the PCX_2–4_C motif of A3A. Deletion of these amino acids in the A3A/ΔWG mutant slightly reduced *in vitro* deaminase activity (Figure 6). The variable region VS1 is situated immediately upstream of the H-X-E-X_23–28_-P-C-X_4_-C conserved motif, and replacement of A3A sequences with those from A3G caused a decrease in deaminase activity. Substitution of the VS1 region in A3G with sequences from A3A increased deaminase activity compared to wild-type A3G (Figure S6). This suggests that the VS1 region (residues 60–67) may contribute to the increased enzymatic activity of A3A [14],[50]. Interestingly, the VS1 region in located in the active center loop 3 of the C-terminal domain of A3G and disruption of this loop results in greatly impaired A3G deaminase activity [55]. In the variable region VS2, substitution of A3A amino acids with those from A3G also decreased deamination, suggesting that this region is important for catalytic activity. This observation is supported by the decrease in deaminase activity observed for the reciprocal A3G mutants (A3G/YDP and A3G/YDPLYK) (Figure S6). Together these results indicate that regions outside of the active site contribute to catalytic activity of A3A. In support of our observations, a recent mutagenesis study of A3G also suggested that C-terminal residues (residues 276–384) are important for deaminase activity [60].

Multiple mechanisms have been proposed to explain the deaminase-independent inhibition of retroelements and HBV by APOBEC3G [35],[39]. In the case of retroviruses, the APOBEC3 proteins have been suggested to inhibit RT, prevent accumulation of reverse transcripts and viral cDNA in target cells, and block integration [37],[42],[44],[45]. Biochemical studies have shown that purified recombinant A3G inhibited RT-catalyzed DNA elongation *in vitro* and this was independent of its deaminase activity [63]. A3G can inhibit HBV in the absence of extensive editing [12], and has been suggested to be due to inhibition of early steps in viral reverse transcription and strand elongation [48]. AAV inhibition differs from these other systems because it does not involve an RT-mediated step or any RNA substrates. Since A3A also inhibits MVM replication and does not affect production of viral proteins [14], we favor the idea that A3A inhibits parvovirus replication through a direct interaction with the viral DNA or the replication machinery. Binding to ssDNA by A3G is proposed to inhibit RT processivity [21],[63], and binding of A3A to ssDNA in the parvovirus genome could physically block movement of the DNA polymerase along the viral template. Although this inhibition would be independent of catalytic activity, amino acids in the active site may be required for efficient nucleic acid binding, explaining the loss of antiviral activity for mutants such as E72Q or C106S. Preliminary results suggest that the F75L mutant retains its ability to bind nucleic acid (data not shown). Potential binding sites could be the viral ITR or the ssDNA/dsDNA junction, which may reduce Rep binding and inhibit DNA synthesis. A3A is not found in high molecular weight complexes that have been reported to modulate A3G activity [64],[65], however we cannot exclude the possibility that AAV inhibition might be mediated through A3A interactions with AAV Rep or cellular proteins that are required for AAV replication [66]. It will be interesting to investigate whether recombinant A3A can block viral DNA replication in an *in vitro* replication assay where we could test the direct activity of A3A on AAV replication [67]. This approach, however, is currently limited by the requirement for purified recombinant A3A and its mutants.

Although 293 cells are a standard system used to study parvovirus replication, it remains unclear whether endogenous A3A restricts parvovirus infection *in vivo*. It is unknown which cells represent the primary site of AAV infection and replication *in vivo*. It has been shown that expression of APOBEC3 proteins is induced in response to interferon-α (IFNα) [68]. The levels of A3A that achieve inhibition of AAV in transfected cells are within the range of endogenous A3A levels detected in peripheral blood mononuclear cells (PBMCs) and macrophages activated with IFNα (Figure S7). Therefore, it would be interesting to test whether cells refractory to parvovirus replication will allow AAV replication when endogenous A3A levels are reduced. In addition to parvoviruses, A3A is active against LINE1 and other retrotransposons [14],[50],[51],[64],[69]–[71] where hypermutation is not detected. It will be informative to determine whether this occurs by a mechanism similar to parvovirus inhibition. In summary, our study demonstrates that the DNA cytidine deaminase activity of A3A is not required for inhibition of parvovirus replication. The combination of the single-domain cytidine deaminase A3A with the simple model system of parvovirus replication provides a valuable tool to uncover new mechanisms for the antiviral activity of the APOBEC3 proteins.

## Materials and Methods

### Cell Lines

293T, HeLa, A9 and human osteosarcoma U2OS cell lines were purchased from the American Tissue Culture Collection. Cells were grown as monolayers in Dulbecco's modified Eagle's medium (DMEM) supplemented with 10% fetal bovine serum and antibiotics at 37°C in a humid atmosphere containing 5% CO_2_.

### Expression Plasmids

Expression plasmids encoding cDNAs for A3A (NM_145699) and A3G (NM_021822) and the A3A mutants H70R, E72Q, and C106S in the pcDNA3.1 (+) vector with a hemagglutinin (HA) tag at the C-terminus have been previously described [14]. New A3A and A3G mutants were generated by site-directed mutagenesis using the QuikChange kit (Stratagene) (Table S1). The truncated form of A3G (A3G-CT, residues 197-384) was generated by PCR amplification of its C-terminus. Plasmids expressing AAV Rep/Cap proteins (pXX2) and the adenovirus helper proteins (pXX6) have been described [72]. The rAAV vector plasmid (pACLALuc) consists of the luciferase gene amplified from pGL3basic (Promega) cloned into an ITR-flanked expression cassette under the control of the CMV promoter and the BGH polyadenylation signal (pACLA). The complete ITR-flanked expression cassette in pACLALuc is 4.3 Kb.

### Production of Recombinant AAV

Recombinant AAV production assays were performed as previously described [14],[72]. Briefly, 293T cells were seeded at 0.5×10^6^ cells/well in 6-well plates and the next day were co-transfected with pXX6 (2.25 µg), pXX2 (0.75 µg), pACLALuc (0.75 µg) and APOBEC3 expression vector (1 µg unless otherwise stated) or pcDNA3.1(+) control vector (1 µg). Dose-response titrations maintained the total amount of effector DNA by addition of pcDNA3.1(+). Transfections were performed in duplicate or triplicate using polyethyleneimine (PEI) [73]. Cells were harvested 72 h post-transfection after two washes in ice-cold PBS, and one third of each sample was removed for immuno-blotting. The other two thirds of the cells were used to generate rAAVLuc virus lysates by freeze/thaw cycles followed by centrifugation. Virus lysates were used to transduce 293T cells in 48 wells in triplicate. Transduced cells were incubated with Steady-Glo luciferase substrate reagent (Promega) 48 h post–transduction and lucifierase activity was quantified in triplicate in 96 well Lumiplates (Greiner Bio-One) in a TopCount NXT scintillation and luminescence counter (PerkinElmer). rAAV production experiments are presented as mean+SEM of the relative value (%) of at least three independent experiments, and compared to mock transfections with pcDNA3.1(+).

### Immunoblotting

Immunoblotting was performed essentially as described [14]. Cell pellets from rAAVLuc production assays were lysed in lysis buffer (137 mM NaCl, 2.68 mM KCl, 10.1 mM Na_2_HPO_4_, 1.76 mM KH_2_PO_4_, 1 mM NaO_3_V, 20 mM β-glycerol phosphate, 20 mM NaF, 0.1% NP40, and 0.025% Triton-X 100) supplemented with Complete protease inhibitor cocktail (Roche) for 30 min on ice. The lysates were clarified by centrifugation at 10,000×g for 20 min. Protein concentrations from whole cell lysates were quantified by BCA assay (Bio-Rad), and 20 µg of protein was loaded per well onto polyacrylamide gels. Proteins were separated in 4–12% or 12% Acrylamide Bis-Tris NuPage gels in MOPS buffer (Invitrogen) and transferred onto Hybond nitrocellulose membranes (Amersham Biosciences). Membranes were probed with anti-HA 16b12 monoclonal antibody (mAb) (Covance) and anti-Ku86 mAb (Santa Cruz). Bound antibody was detected by incubation with goat anti-mouse antibody conjugated to horseradish peroxidase (Jackson ImmunoResearch), and the bands were visualized with enhanced chemiluminiscence reagent (ECL Western Lightning Kit, PerkinElmer) followed by autoradiography.

### Immunofluorescence

APOBEC3 protein localization was determined by indirect immunoflorescence [14]. U2OS or HeLa cells were grown on glass coverslips in 24 well plates and transfected with 0.8 µg APOBEC3 expression vector using Lipofectamine 2000 (Invitrogen). After 36–48 h, cells were washed with PBS, fixed with 3% paraformaldehyde for 20 min and extracted with 0.5% Triton X-100 in PBS for 10 min. Cells were incubated with 3% BSA for 30 min, followed by incubation with anti-HA mAb 16b12 (1∶2000). A 1∶2000 dilution of goat anti-mouse conjugated Alexa Fluor 568 (Invitrogen) and DAPI (Sigma Aldrich) in 3% BSA in PBS was added to cells and samples were incubated for 1 h at room temperature. The coverslips were mounted in Fluoromount-G (Southern Biotech) and cells were visualized by fluorescence microscopy (Diaphot 300 inverted microscope, Nikon). For AAV replication U2OS cells were seeded on glass coverslips and transfected with APOBEC3 expression vector, and infected 16 h post-transfection with wild-type AAV and/or adenovirus. After 24 hr, the cells were fixed and stained with anti-HA and anti-Rep antibodies and DAPI as described above. Rep from the input virus is undetectable in this assay, so positive Rep staining is indicative of AAV replication.

### Southern Blot Hybridization

Low molecular weight AAV episomal DNA was analyzed by Southern hybridization with a ^32^P-labeled luciferase probe as previously described [74]. Briefly, 293T cells grown in 6 well tissue culture plates to 95% confluency were co-transfected with plasmids for rAAV production using Lipofectamine 2000 following the manufacturer's protocol. The following effector plasmids were included: pcDNA3.1(+) (1 µg), A3G (1ug), A3A (1 µg, 0.1 µg, 0.01 µg and 0.001 µg), F75L (1 µg) and F95L (1 µg). After 48 h, the cells were collected and washed in PBS. One third of each sample was removed for immunoblotting. DNA was isolated from pellets by a modified HIRT protocol [74] and digested with *Dpn*I (New England Biolabs) to remove input plasmid. DNA was processed by gel electrophoresis on a 1% agarose gel in TAE buffer. The pACLALuc plasmid was digested with *Sma*I as control. The gel was depurinated in 0.2 M HCl, denatured in 1 M NaCl, 0.5 M NaOH, and neutralized in 0.5 M Tris pH 7.5, 1.5 M NaCl. DNA was then transferred to a Hybond XL membrane (Amersham Biosciences) and UV-cross linked. The membrane was hybridized with a ^32^P labeled luciferase probe generated by PCR using the primers described in Table S1 and labeled with ^32^P dCTP using Radivue II labeling kit (Amersham), and visualized in a FLA-5100 phosphorimager (Fuji).

Southern blot replication assays of wild-type MVM and MVM-Δ*Bgl*II were performed as previously described [75].

### *In vitro* Deaminase Assays

293T cells were seeded at 5×10^5^ cells/well in 6-well plates and transfected after one day with 3 µg of APOBEC3 expression vector. Two days post-transfection, the cells were rinsed twice with cold PBS and lysed for 30 min on ice in lysis buffer (50 mM Tris, pH 8.0, 40 mM KCl, 50 mM NaCl, 5 mM EDTA, 0.1% Triton X-100, 10 mM DTT). Lysates were clarified by centrifugation at 13,000×g for 10 min and pre-cleared with 50 µl of High flow protein-G-Sepharose (Amersham). The lysate was incubated with anti-HA mAb 3F10 (Roche) for 2 h at 4°C. The lysate-antibody was then incubated with High flow protein-G-Sepharose for 1-2 h at 4°C. The resin was washed three times with lysis buffer. One-fifth of the resin was removed for immunoblot analysis and the remainder was washed once with deaminase reaction buffer (40 mM Tris, pH 8.0, 10% glycerol, 40 mM KCl, 50 mM NaCl, 5 mM EDTA, and 1 mM DTT). PAGE purified deoxyoligonucleotide (Table S1) was 5′-end ^32^P labeled and added into 20 µl of deaminase reaction buffer. The reaction was incubated at 37°C for 20 h, stopped by heating to 90°C for 5 min, cooled on ice, and then centrifuged to collect the resin at the bottom of tube. The supernatant was incubated with uracil DNA glycosylase (New England Biolabs) in buffer containing 20 mM Tris, pH 8.0, 1 mM DTT for 1 h at 37°C and treated with 150 mM NaOH for 1 h at 37°C. The samples were incubated at 95°C for 5 min, 4°C for 2 min and separated by 15% TBE/urea-PAGE. The gel was dried, exposed to a phosphorimager screen and analyzed using a FLA-5100 scanner (Fuji).

For *in vitro* synthesis of A3A and mutants we employed the TNT Coupled Wheat Germ Extract System (Promega) using T7 polymerase. Translation reactions were performed with non-labeled amino acids following the manufacturer's protocol in 50 µl final volume that included 1 µg of pcDNA3.1(+) plasmid encoding APOBEC3. Reactions were incubated for 90 min at 30°C. After incubation, 450 µl of TritonX-100 buffer (50 mM Tris, pH 8.0, 40 mM KCl, 50 mM NaCl, 5 mM EDTA, 0.1% Triton X-100, 10 mM DTT) were added to the reactions and used to analyze deaminase activity after immunoprecipitation as described above.

In order to measure deaminase activity directly from IVT reactions, after 90 min incubation at 30°C translation reactions were centrifuged at 10,000×g for 1 min. An aliquot of supernatant (5 µl) was removed for immunoblot analysis. The reaction mixture (15 µl of supernatant) was incubated with 5′-end ^32^P labeled deoxyoligonucleotide in 30 µl of deaminase reaction buffer and assayed for deaminase activity following the same procedure described above.

### FRET-Based Deaminase Activity Assay from Cell Lysates

To quantify deaminase activity from cell lysates, we used a modification of the FRET-based protocol described by Thielen *et al.* [54]. 293T cells were seeded at 1×10^6^ cells/well in 6 cm plates and a day later transfected with 8 µg of APOBEC3 expression vector. After 36 to 48 h, cells were resuspended in lysis buffer (200 µl) and lysates were obtained as described above. Cell lysate (10 µl) was mixed with 70 µl of FRET deaminase buffer (40 mM Tris, pH 8.0, 40 mM KCl, 50 mM NaCl, 5 mM EDTA) containing 10 pmol of dual-labeled probe (Table S1) and 0.4 units of UDG (New England Biolabs). Reactions were incubated at 37°C for 90 min followed by addition of 4 µl of 4N NaOH and incubation at 37°C for 30 min. Reactions were neutralized with 4 µl of 4N HCl and 36 µl of 1 M Tris-HCl (pH 8). 6FAM fluorescence was measured at 25°C in an Mx30005P (Stratagene). Two-fold serial dilutions of each lysate were analyzed in duplicate. Fluorescence detected in 293T cells transfected with pcDNA3.1 (mock) was substracted from all samples and deaminase activity is shown as relative fluorescence units (RFU).

### Detection of Endogenous A3A

Human monocytes were purified from leukocyte enriched blood samples (New York Blood Center) using CD14+ magnetic beads (Miltenyi Biotec) according to manufacturer instructions. The CD14^+^ monocytes were cultured with 50 ng/ml GM-CSF (Invitrogen) for 7 days in order to differentiate them into macrophages. The monocyte-derived macrophages were plated in 12 well plates at 10^6^ cells per well and cultured with or without 2000U of Universal Type I Interferon (PBL Biomedical Laboratories) for 20 hours. The cells were then collected in lysis buffer. Cell lysates from human peripheral blood mononuclear cells (PBMCs) were obtained from F. Chisari (The Scripps Research Institute) [76]. PBMCs were treated with 1000U of IFN-α or kept untreated for 24 hrs before cell lysates were generated. Lysates of PBMCs (60 µg) and macrophages (30 µg) of each sample were run on 4–12% or 12% Bis-Tris gels and analyzed by immunoblotting as described above. Anti-A3A (raised against an N-terminal peptide) (1∶250 dilution) or anti-recombinant A3A (1∶1000 dilution) polyclonal rabbit sera were used for A3A detection as described.

## Supporting Information

Figure S1 APOBEC3A proteins synthesized from coupled *in-vitro* transcription-translation are active in UDG-dependent deaminase assays. Deaminase activity of A3A and mutant proteins generated by cell transfection and *in-vitro* coupled transcription-translation (IVT) was analyzed in UDG-dependent deaminase assays. (A) 293T cells were transfected with plasmids for A3A and mutants. Cells were harvested at 48 hrs post-transfection, and lysates were subject to immunoprecipitation (IP) with anti-HA antibody (3F10). 4/5 of the IP was incubated with a 5′-end ^32^P labeled T_28_TCAT_28_ deoxyoligonucleotide and tested in UDG-dependent deaminase assays. Arrows indicate substrate deoxyoligonucleotide and cleaved deaminated product. Bottom panel shows an immunoblot corresponding to 1/5 of the IP analyzed with an anti-HA antibody (16B12). Asterisks indicate bands corresponding to IgG light chain. A3A truncations are: TruncA (aa 1–145), TruncB (aa 1–165), and TruncC (aa 53–199). (B) Wild-type and mutant A3A proteins were synthesized by IVT as described in Methods. pcDNA3.1(+) was included in IVTs as mock. A3A proteins were immunoprecipitated with 3F10 antibody and tested in the UDG-dependent deaminase assay. Immunoprecipitated A3A from transfected cells was included as a control. Middle panel shows 1/5 of the IP protein analyzed by immunoblotting with 16B12 antibody. Bottom panel shows immunoblotting of lysates to demonstrate that equal amounts of protein were generated by IVT. (C) Wild-type and mutant A3A proteins were tested directly from IVT reactions for deaminase activity. Bottom panel shows an immunoblot of 1/5 of the IVT-synthesized proteins loaded into the deaminase reactions.
(1.92 MB PDF)

Figure S2 Lack of deaminase activity for F75L is not due to modified target sequence preference and is supported by the lack of deamination measured by FRET. (A) To rule out differences in target sequence preference, A3A and F75L were tested in UDG-dependent deaminase assays against 16 different target sites (Table S1). Target sequence preference of A3A and F75L was determined on a panel of four ^32^P-labeled deoxyoligonucleotide substrates, each containing four target sites. The -1 base is shown at the top of each lane and the +1 base is shown on the left. The structure of the cleaved products is shown on the right. (B) Quantification of deaminase acitivity by FRET in cell lysates obtained from 293T cells transfected with A3A, E72Q, F75L, F95L and C106S. Cell lysates were incubated with a dual-labeled probe containing the CCCG target sequence (Table S1). The TAMRA fluorophore quenches emission by the 6FAM fluorophore. After deamination and treatment with UDG and high pH, 6FAM fluorescence emission can be detected from the cleaved probe. Two-fold serial dilutions of each lysate were analyzed by duplicate and represented as mean±SEM. Results show that deaminase activity is detected in cell lysates obtained from cells transfected with A3A and F95L, while deaminase activity of E72Q, F75L and C106S is not distinguishable from the background level. Bottom panel shows an immunoblot of cell lysates.
(2.23 MB PDF)

Figure S3 Localization of A3A/A3G chimeras. Immunofluorescence to detect localization of HA-tagged APOBEC3 and chimeric proteins (red) expressed by transfection in U2OS cells. Cell nuclei were detected by staining with DAPI (blue). (A) Localization of wild-type APOBEC3 proteins and A3A/A3G chimeras. (B) Localization of A3A mutants with sequences incorporated from A3G.
(9.29 MB PDF)

Figure S4 Inhibition of rAAV production by A3A/A3G chimeras in the VS1 region. Dose-response for A3A and mutant proteins in the rAAV production assay. Virus production was assessed by transduction of target cells and quantitation by luciferase assay. Panels below show immunoblots for APOBEC3 (HA) and Ku86 proteins in transfected cells.
(0.39 MB PDF)

Figure S5 Dose-response antiviral activity of A3G-CT/KNLLCGFY. Comparison of A3A and A3G-CT/KNLLCGFY antiviral activity over a dose-response in rAAV production assays. Comparable levels of A3A and A3G-CT/KNLLCGFY resulted in ∼95% and ∼50% inhibition respectively. Bottom panels show immunoblots for A3A and A3G-CT/KNLLCGFY (HA), and Ku86 protein as a loading control. The asterisks indicate that the inhibition with A3G-CT/KNLLCGFY was statistically significant (p<0.001) when compared to mock by Student *t* test.
(0.53 MB PDF)

Figure S6 Chimeric A3G proteins with sequences replaced with VS1 and VS2 from A3A. (A) Immunofluorescence to detect localization of HA-tagged APOBEC3 and chimeric proteins (red) expressed by transfection in U2OS cells. Cell nuclei were detected by staining with DAPI (blue). (B) *In vitro* deamination assay. Proteins were immunoprecipitated from transfected cells and incubated with the radiolabeled substrate in the standard assay. Arrows indicate the substrate and deaminated product. The panel below shows an immunoblot to detect proteins in immunoprecipitates. Arrows indicate bands corresponding to APOBEC3 proteins. Asterisk indicates bands corresponding to IgG light chain. (C) Production of rAAV in the presence of APOBEC3 proteins. Virus production was assessed by transduction of target cells and quantitation by luciferase assay. Immunoblots show similar expression levels for APOBEC3 proteins (HA) in transfected cells. Ku86 served as a loading control.
(3.93 MB PDF)

Figure S7 Antiviral activity of A3A is achieved with physiological levels of transfected A3A. (A) Antiviral activity of A3A was analyzed in a rAAVLuc production experiment over a dose response. Expression levels of A3A were analyzed by immunoblotting using an anti-HA antibody (top blot), and compared to endogenous levels of A3A expressed in human PBMCs incubated with IFNα. A3A was detected using a rabbit polyclonal antisera raised against an N-terminal A3A specific peptide (bottom blot). Arrow indicates bands corresponding to A3A. Background band below A3A is indicated with an asterisk. (B) In panel B the expression levels of A3A are compared to endogenous levels of A3A induced in human macrophages in response to IFNα. A3A was detected with polyclonal rabbit serum generated to recombinant A3A protein.
(1.09 MB PDF)

Table S1 Oligonucleotides used in this study. Sequences corresponding to the oligonucleotides used in this study. Name, template and type of experiments are indicated for each oligonucleotide. D.A. (deaminase assays). N/A (not applicable).
(0.08 MB DOC)
