# Supplementary material for: Deaminase-Independent Inhibition of Parvoviruses by the APOBEC3A Cytidine Deaminase
Source: PLoS Pathog. 2009 May 22;5(5):e1000439. doi: 10.1371/journal.ppat.1000439 (PMC2678267; doi:10.1371/journal.ppat.1000439)
Supplement: Figure S1 — APOBEC3A proteins synthesized from coupled in-vitro transcription-translation are active in UDG-dependent deaminase assays. Deaminase activity of A3A and mutant proteins generated by cell transfection and in-vitro coupled transcription-translation (IVT) was analyzed in UDG-dependent deaminase assays. (A) 293T cells were transfected with plasmids for A3A and mutants. Cells were harvested at 48 hrs post-transfection, and lysates were subject to immunoprecipitation (IP) with anti-HA antibody (3F10). 4/5 of the IP was incubated with a 5′-end 32P labeled T28TCAT28 deoxyoligonucleotide and tested in UDG-dependent deaminase assays. Arrows indicate substrate deoxyoligonucleotide and cleaved deaminated product. Bottom panel shows an immunoblot corresponding to 1/5 of the IP analyzed with an anti-HA antibody (16B12). Asterisks indicate bands corresponding to IgG light chain. A3A truncations are: TruncA (aa 1–145), TruncB (aa 1–165), and TruncC (aa 53–199). (B) Wild-type and mutant A3A proteins were synthesized by IVT as described in Methods. pcDNA3.1(+) was included in IVTs as mock. A3A proteins were immunoprecipitated with 3F10 antibody and tested in the UDG-dependent deaminase assay. Immunoprecipitated A3A from transfected cells was included as a control. Middle panel shows 1/5 of the IP protein analyzed by immunoblotting with 16B12 antibody. Bottom panel shows immunoblotting of lysates to demonstrate that equal amounts of protein were generated by IVT. (C) Wild-type and mutant A3A proteins were tested directly from IVT reactions for deaminase activity. Bottom panel shows an immunoblot of 1/5 of the IVT-synthesized proteins loaded into the deaminase reactions. (1.92 MB PDF) [file ppat.1000439.s001.pdf]

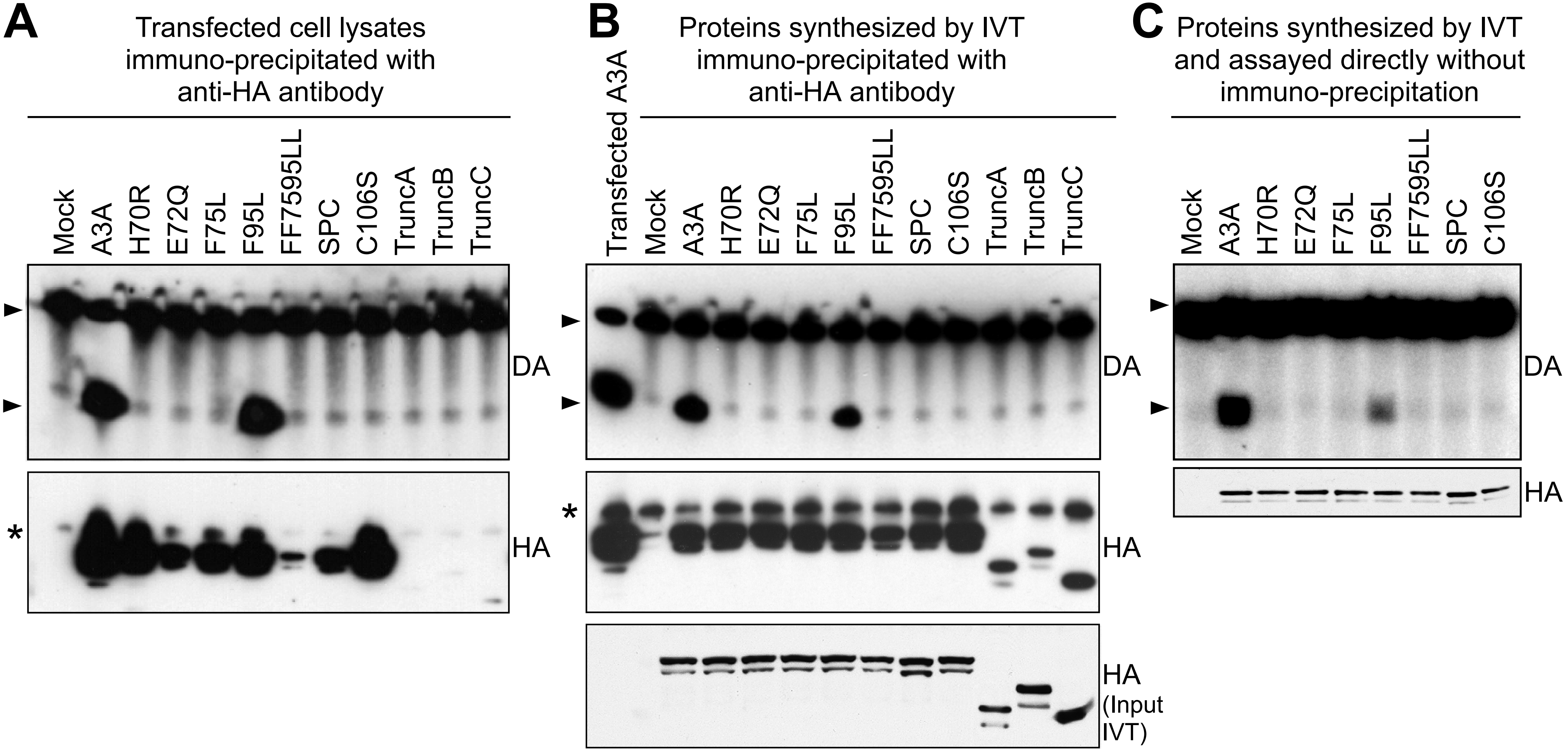
A
Transfected cell lysates immuno-precipitated with anti-HA antibody
Mock
A3A
H70R
E72Q
F75L
F95L
FF7595LL
SPC
C106S
TruncA
TruncB
TruncC
DA
HA
*
B
Proteins synthesized by IVT immuno-precipitated with anti-HA antibody
Transfected A3A
Mock
A3A
H70R
E72Q
F75L
F95L
FF7595LL
SPC
C106S
TruncA
TruncB
TruncC
DA
HA
*
HA (Input IVT)
C
Proteins synthesized by IVT and assayed directly without immuno-precipitation
Mock
A3A
H70R
E72Q
F75L
F95L
FF7595LL
SPC
C106S
DA
HA
