# Supplementary figures and images for: Deaminase-Independent Inhibition of Parvoviruses by the APOBEC3A Cytidine Deaminase
Source: PLoS Pathog. 2009 May 22;5(5):e1000439. doi: 10.1371/journal.ppat.1000439 (PMC2678267; doi:10.1371/journal.ppat.1000439)

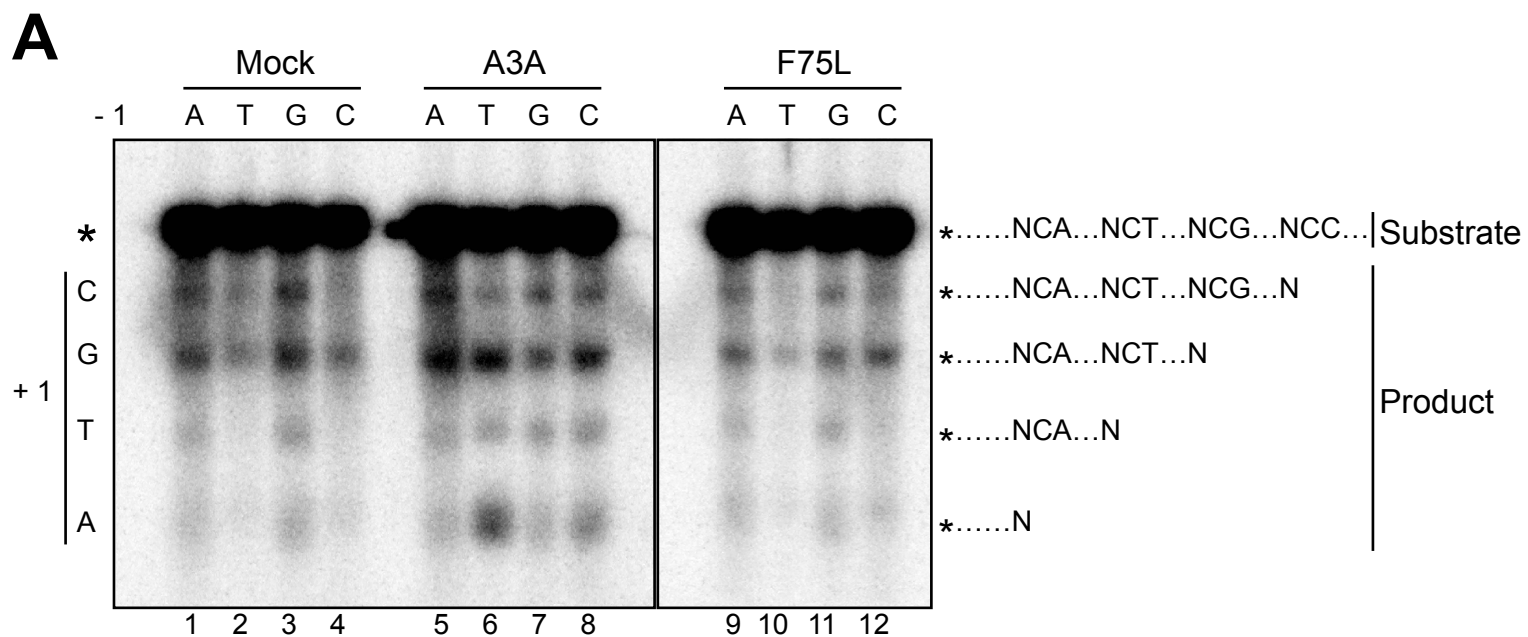

**B**

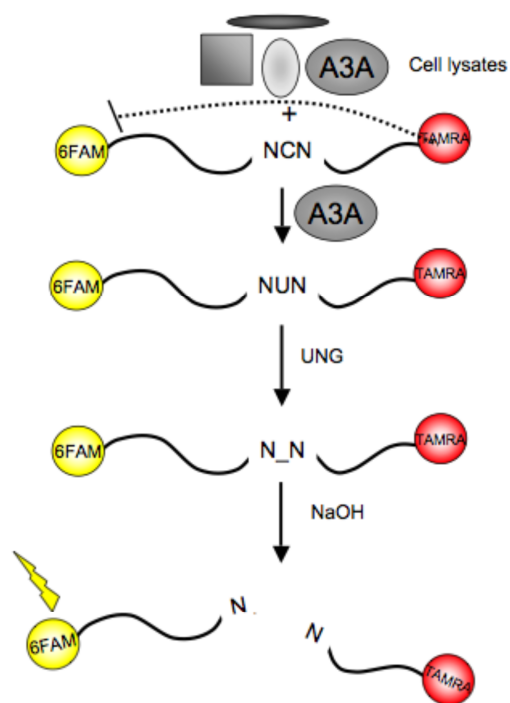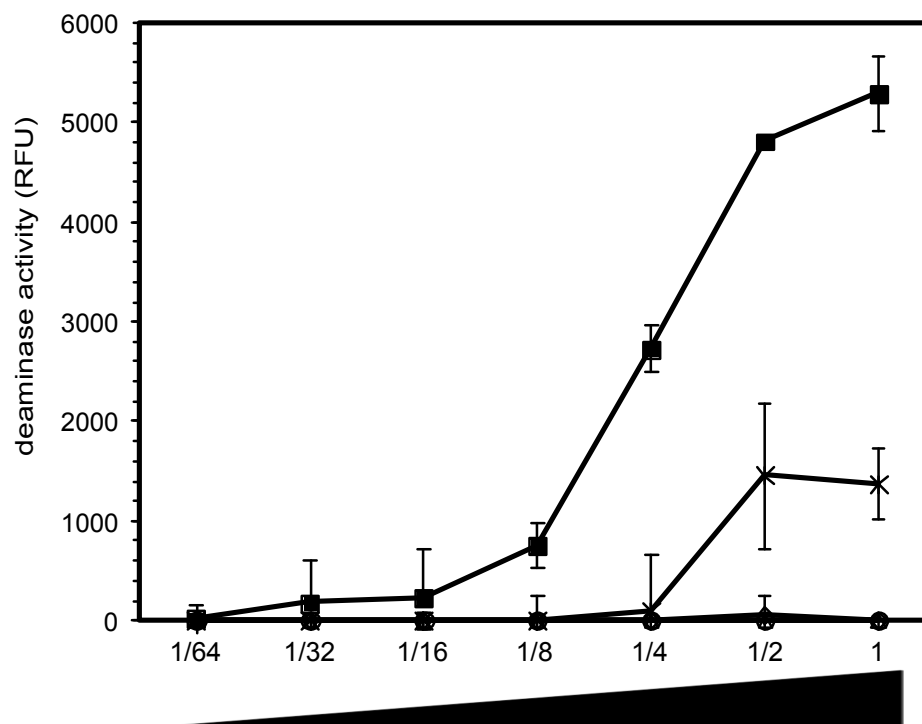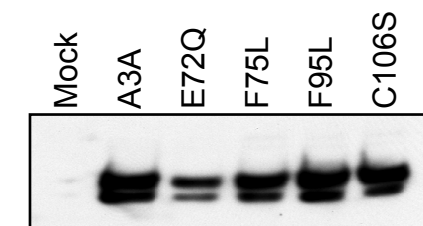

Supplement: Figure S2 — Lack of deaminase activity for F75L is not due to modified target sequence preference and is supported by the lack of deamination measured by FRET. (A) To rule out differences in target sequence preference, A3A and F75L were tested in UDG-dependent deaminase assays against 16 different target sites (Table S1). Target sequence preference of A3A and F75L was determined on a panel of four 32P-labeled deoxyoligonucleotide substrates, each containing four target sites. The -1 base is shown at the top of each lane and the +1 base is shown on the left. The structure of the cleaved products is shown on the right. (B) Quantification of deaminase acitivity by FRET in cell lysates obtained from 293T cells transfected with A3A, E72Q, F75L, F95L and C106S. Cell lysates were incubated with a dual-labeled probe containing the CCCG target sequence (Table S1). The TAMRA fluorophore quenches emission by the 6FAM fluorophore. After deamination and treatment with UDG and high pH, 6FAM fluorescence emission can be detected from the cleaved probe. Two-fold serial dilutions of each lysate were analyzed by duplicate and represented as mean±SEM. Results show that deaminase activity is detected in cell lysates obtained from cells transfected with A3A and F95L, while deaminase activity of E72Q, F75L and C106S is not distinguishable from the background level. Bottom panel shows an immunoblot of cell lysates. (2.23 MB PDF) [file ppat.1000439.s002.pdf]

**A**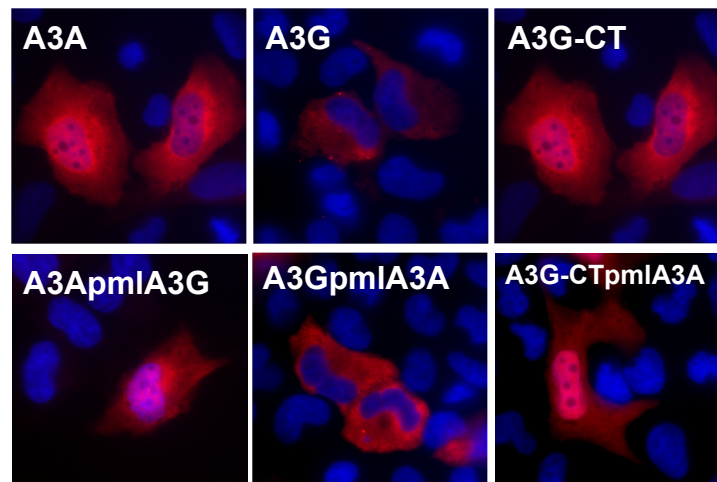**B**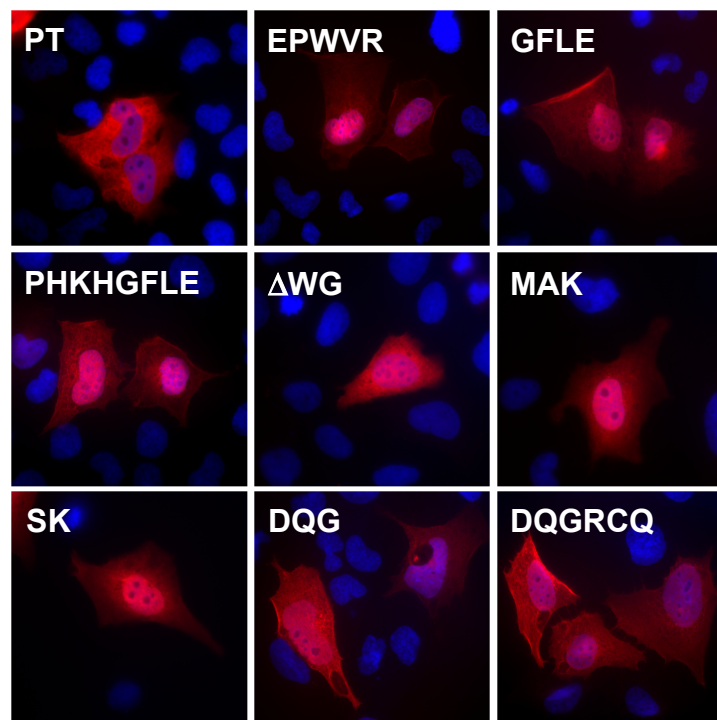

Supplement: Figure S3 — Localization of A3A/A3G chimeras. Immunofluorescence to detect localization of HA-tagged APOBEC3 and chimeric proteins (red) expressed by transfection in U2OS cells. Cell nuclei were detected by staining with DAPI (blue). (A) Localization of wild-type APOBEC3 proteins and A3A/A3G chimeras. (B) Localization of A3A mutants with sequences incorporated from A3G. (9.29 MB PDF) [file ppat.1000439.s003.pdf]

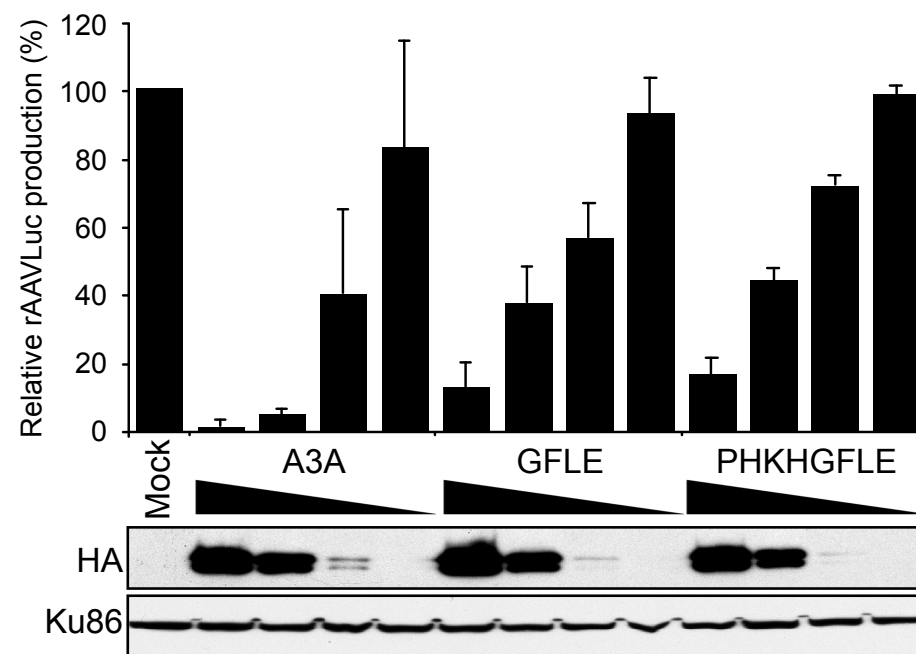

Supplement: Figure S4 — Inhibition of rAAV production by A3A/A3G chimeras in the VS1 region. Dose-response for A3A and mutant proteins in the rAAV production assay. Virus production was assessed by transduction of target cells and quantitation by luciferase assay. Panels below show immunoblots for APOBEC3 (HA) and Ku86 proteins in transfected cells. (0.39 MB PDF) [file ppat.1000439.s004.pdf]

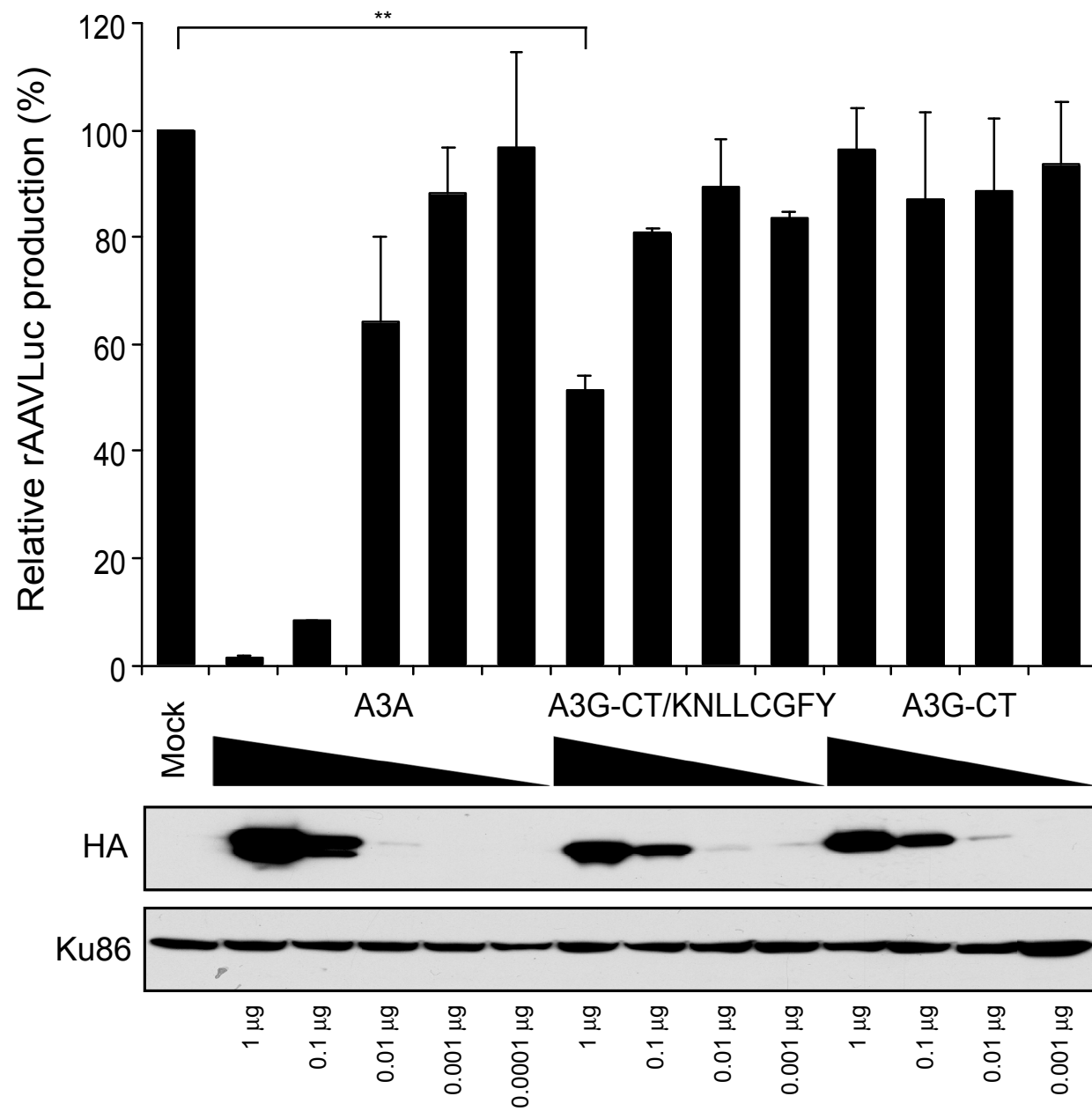

Supplement: Figure S5 — Dose-response antiviral activity of A3G-CT/KNLLCGFY. Comparison of A3A and A3G-CT/KNLLCGFY antiviral activity over a dose-response in rAAV production assays. Comparable levels of A3A and A3G-CT/KNLLCGFY resulted in ∼95% and ∼50% inhibition respectively. Bottom panels show immunoblots for A3A and A3G-CT/KNLLCGFY (HA), and Ku86 protein as a loading control. The asterisks indicate that the inhibition with A3G-CT/KNLLCGFY was statistically significant (p<0.001) when compared to mock by Student t test. (0.53 MB PDF) [file ppat.1000439.s005.pdf]

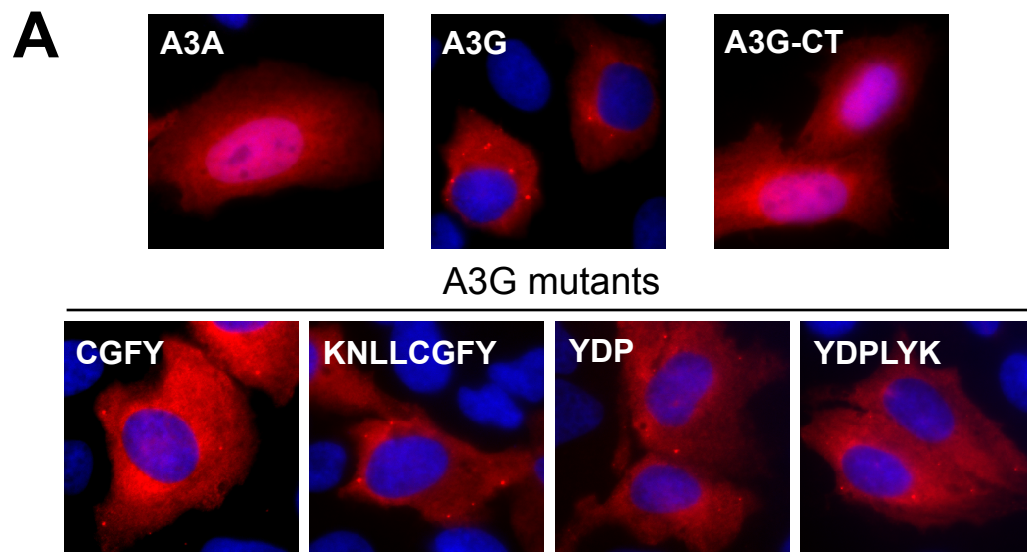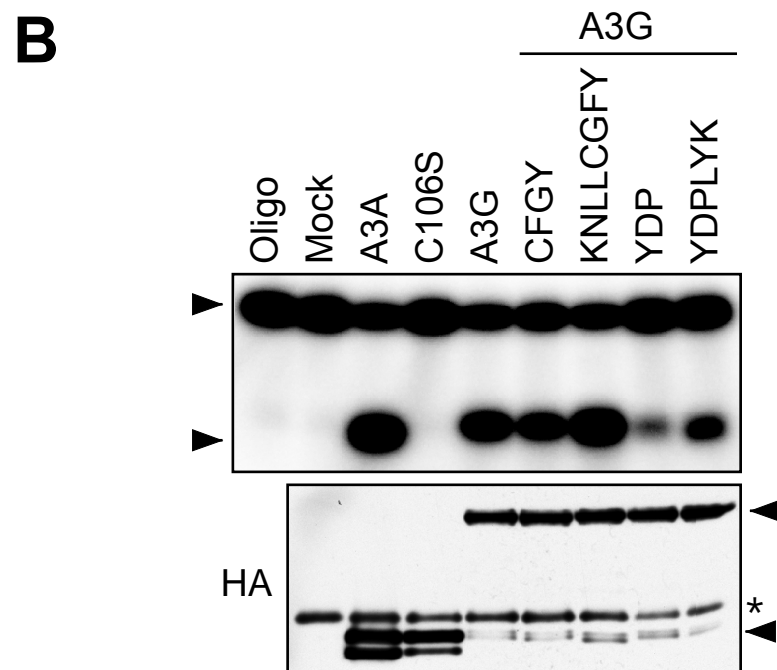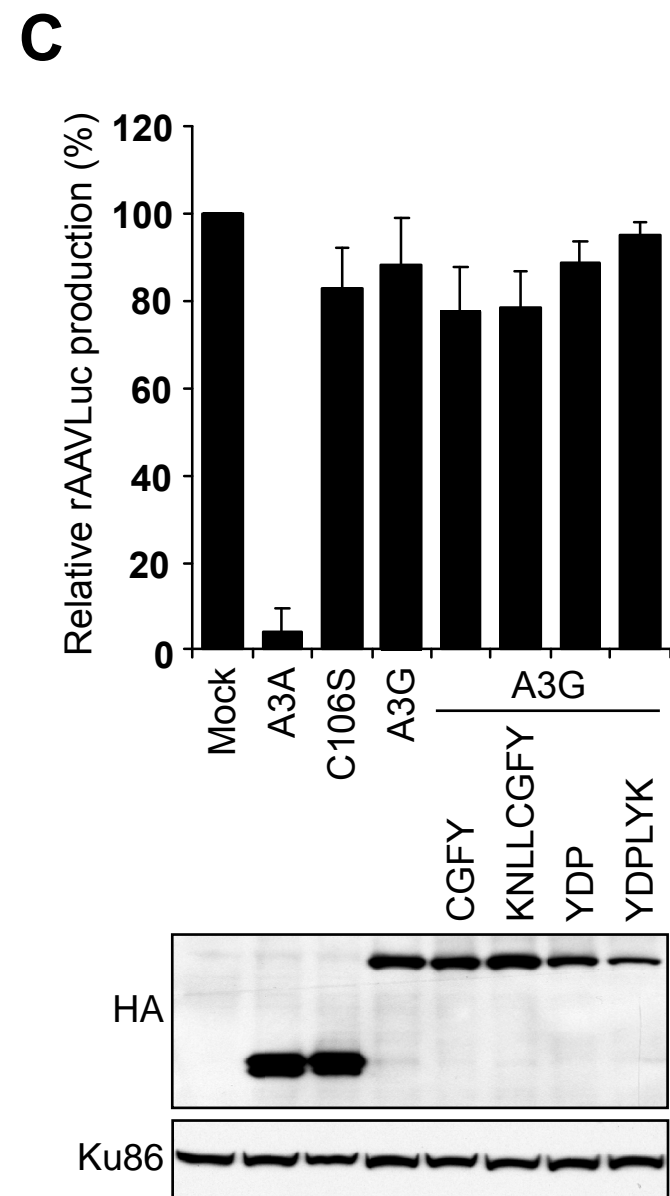

Supplement: Figure S6 — Chimeric A3G proteins with sequences replaced with VS1 and VS2 from A3A. (A) Immunofluorescence to detect localization of HA-tagged APOBEC3 and chimeric proteins (red) expressed by transfection in U2OS cells. Cell nuclei were detected by staining with DAPI (blue). (B) In vitro deamination assay. Proteins were immunoprecipitated from transfected cells and incubated with the radiolabeled substrate in the standard assay. Arrows indicate the substrate and deaminated product. The panel below shows an immunoblot to detect proteins in immunoprecipitates. Arrows indicate bands corresponding to APOBEC3 proteins. Asterisk indicates bands corresponding to IgG light chain. (C) Production of rAAV in the presence of APOBEC3 proteins. Virus production was assessed by transduction of target cells and quantitation by luciferase assay. Immunoblots show similar expression levels for APOBEC3 proteins (HA) in transfected cells. Ku86 served as a loading control. (3.93 MB PDF) [file ppat.1000439.s006.pdf]

**A**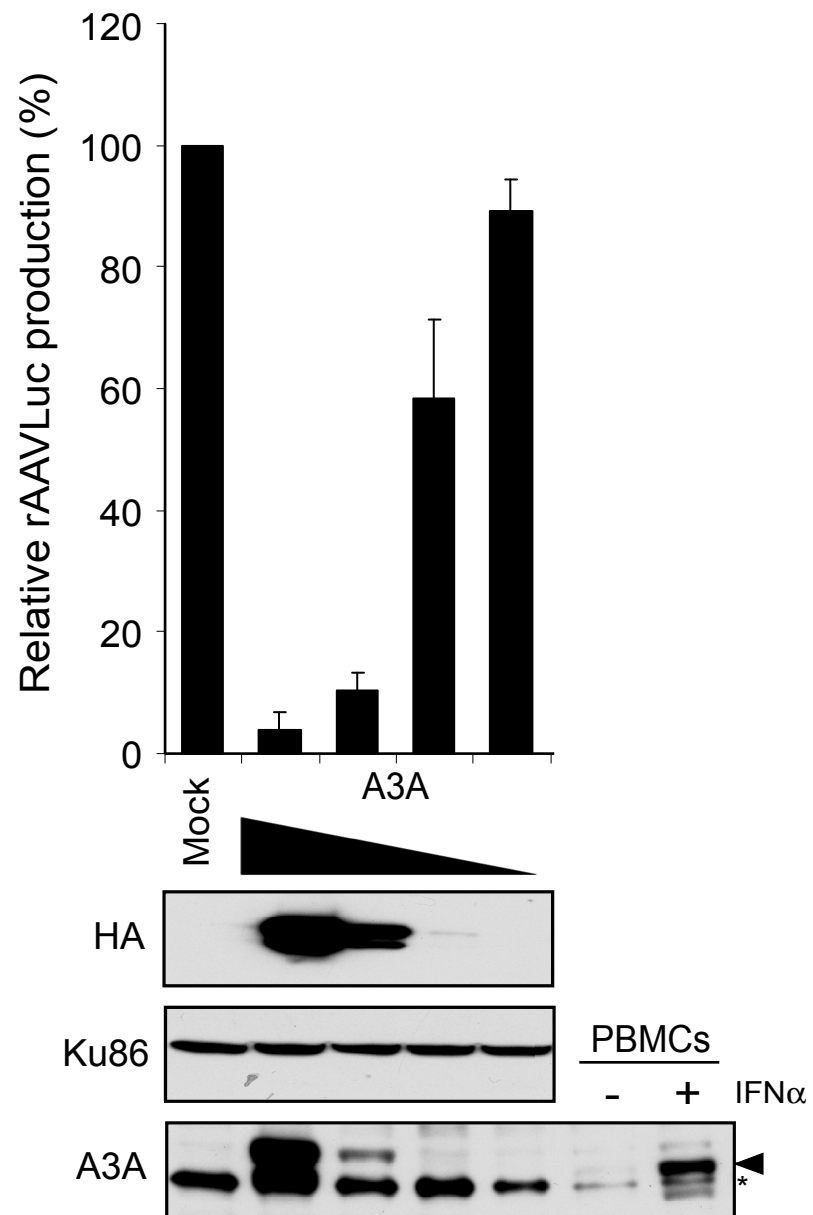**B**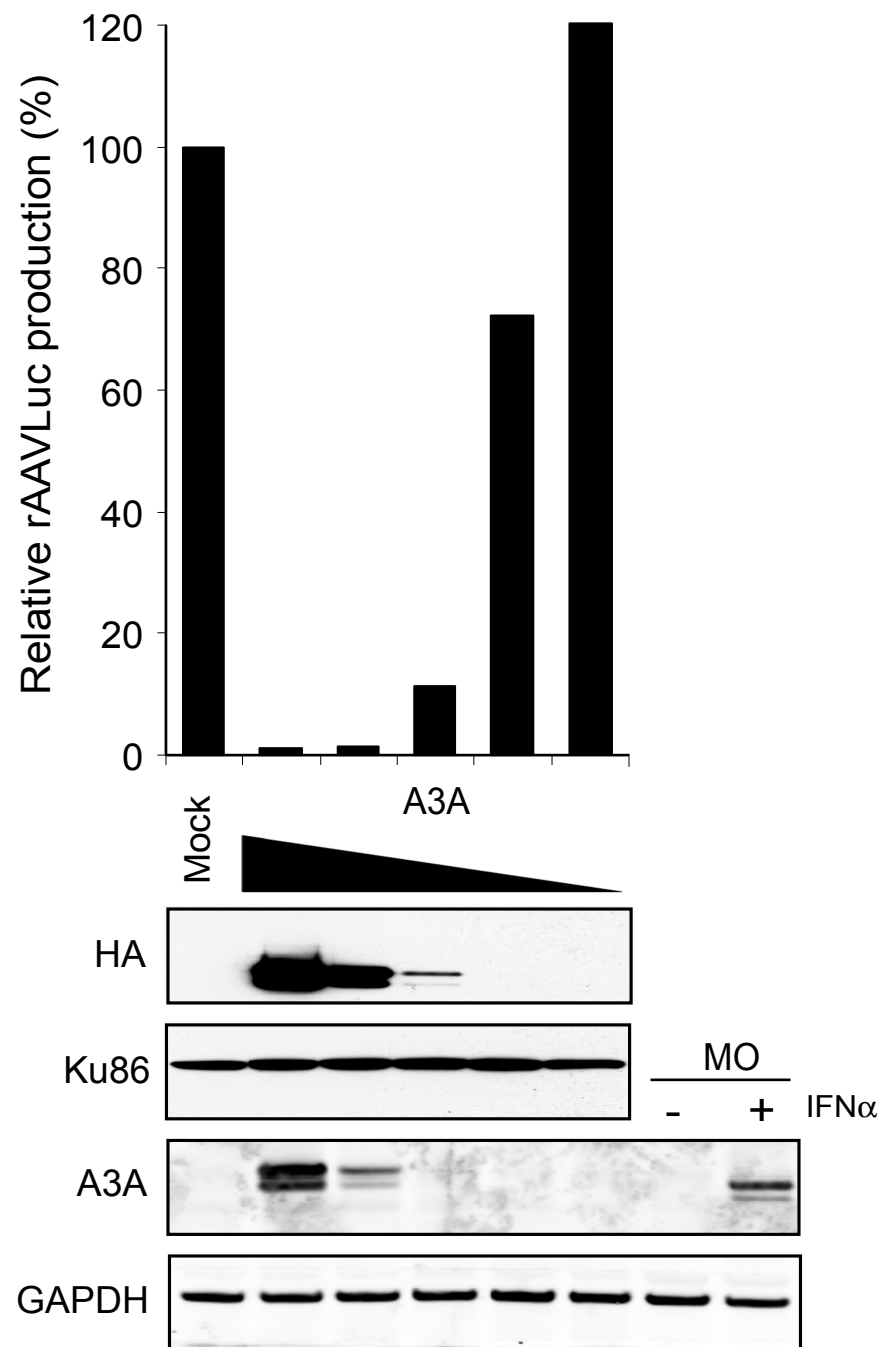

Supplement: Figure S7 — Antiviral activity of A3A is achieved with physiological levels of transfected A3A. (A) Antiviral activity of A3A was analyzed in a rAAVLuc production experiment over a dose response. Expression levels of A3A were analyzed by immunoblotting using an anti-HA antibody (top blot), and compared to endogenous levels of A3A expressed in human PBMCs incubated with IFNα. A3A was detected using a rabbit polyclonal antisera raised against an N-terminal A3A specific peptide (bottom blot). Arrow indicates bands corresponding to A3A. Background band below A3A is indicated with an asterisk. (B) In panel B the expression levels of A3A are compared to endogenous levels of A3A induced in human macrophages in response to IFNα. A3A was detected with polyclonal rabbit serum generated to recombinant A3A protein. (1.09 MB PDF) [file ppat.1000439.s007.pdf]
