# Supplementary material for: Deaminase-Independent Inhibition of Parvoviruses by the APOBEC3A Cytidine Deaminase
Source: PLoS Pathog. 2009 May 22;5(5):e1000439. doi: 10.1371/journal.ppat.1000439 (PMC2678267; doi:10.1371/journal.ppat.1000439)
Supplement: Table S1 — Oligonucleotides used in this study. Sequences corresponding to the oligonucleotides used in this study. Name, template and type of experiments are indicated for each oligonucleotide. D.A. (deaminase assays). N/A (not applicable). (0.08 MB DOC) [file ppat.1000439.s008.doc]

**Table S1: Oligonucleotides used in this study**

| **Oligonucleotide** | **Template** | **Sequence** | **Use** |
| --- | --- | --- | --- |
| F75Lsense | A3A | 5’-GGAACCAGGTCCAATAAGCGCAGCTCCGCATGG-3’ | Mutagenesis |
| F75Las | A3A | 5’-CCATGCGGAGCTGCGCTTATTGGACCTGGTTCC-3’ | Mutagenesis |
| F95Lsense | A3A | 5’-GGCTCCAGGAGATTAACCAAGTGACCCTGTAG-3’ | Mutagenesis |
| F95Las | A3A | 5’-CTACAGGGTCACTTGGTTAATCTCCTGGAGCC-3’ | Mutagenesis |
| SPC99-101AAAs | A3A | 5’-CTTGGTTCATCTCCTGGGCCGCCGCCTTCTCCTGGGGCTGTGCC-3’ | Mutagenesis |
| SPC99-101AAAas | A3A | 5’-GGCACAGCCCCAGGAGAAGGCGGCGGCCCAGGAGATGAACCAAG-3’ | Mutagenesis |
| A3G-CT EcoRI | A3G | 5’-GGAATTCACCATGGATCCACCCACATTC-3’ | PCR |
| BGH Reverse | A3G | 5’-AACTAGAAGGCACAGTCGAGGC-3’ | PCR |
| PTsense | A3A | 5'-AGACACTTGATGGATCCACCCACATTCACTTCCAACTTTAAC-3' | Mutagenesis |
| PTas | A3A | 5'-GTTAAAGTTGGAAGTGAATGTGGGTGGATCCATCAAGTGTCT-3' | Mutagenesis |
| EPWVRsense | A3A | 5'-TCCAACTTTAACAATGAACCTTGGGTCAGAGGAAGGCATAAGACC-3' | Mutagenesis |
| EPWVRas | A3A | 5'-GGTCTTATGCCTTCCTCTGACCCAAGGTTCATTGTTAAAGTTGGA-3' | Mutagenesis |
| DWGsense | A3A | 5'-CTGGAGCCCCTGCTTCTCCTGTGCCGGGGAAGTGCGTG-3' | Mutagenesis |
| DWGas | A3A | 5'-CACGCACTTCCCCGGCACAGGAGAAGCAGGGGCTCCAG-3' | Mutagenesis |
| WGsense | A3G | 5'-CTGGAGCCCCTGCTTCAGTGGGGCCTGTGCCCAGGAAATGGC-3' | Mutagenesis |
| WGas | A3G | 5'-GCCATTTCCTGGGCACAGGCCCCACTGAAGCAGGGGCTCCAG-3' | Mutagenesis |
| MAKsense | A3A | 5’-CTGTGCCGGGGAAATGGCTAAGTTCCTTCAGGAGAACACAC-3’ | Mutagenesis |
| MAKas | A3A | 5’-GTGTGTTCTCCTGAAGGAACTTAGCCATTTCCCCGGCACAG-3’ | Mutagenesis |
| SKsense | A3A | 5'-GAAGTGCGTGCGTTCCTTTCGAAAAACACACACGTGAGAC-3' | Mutagenesis |
| SKas | A3A | 5'-GTCTCACGTGTGTGTTTTTCGAAAGGAACGCACGCACTTC-3' | Mutagenesis |
| GFLEsense | A3A | 5’-CTAAGAATCTTCTCGGTTTCTTAGAAGGCCGCCATG-3’ | Mutagenesis |
| GFLEas | A3A | 5’-CATGGCGGCCTTCTAAGAAACCGAGAAGATTCTTAG-3’ | Mutagenesis |
| PHKHGFLEsense | A3AGFLE | 5’-CAACCAGGCTCCGCATAAACACGGTTTCTTAGAAGGCCGCCATGC-3’ | Mutagenesis |
| PHKHGFLEas | A3AGFLE | 5’-GCATGGCGGCCTTCTAAGAAACCGTGTTTATGCGGAGCCTGGTTG-3’ | Mutagenesis |
| DQGsense | A3A | 5’-CTGCCCGCATCTATGATGACCAAGGCCTATATAAGGAG-3’ | Mutagenesis |
| DQGas | A3A | 5’-CTCCTTATATAGGCCTTGGTCATCATAGATGCGGGCAG-3’ | Mutagenesis |
| DQGRCQsense | A3ADQG | 5’-GCATCTATGATGACCAAGGCCGATGTCAGGAGGCGCTGC-3’ | Mutagenesis |
| DQGRCQas | A3ADQG | 5’-GCAGCGCCTCCTGACATCGGCCTTGGTCATCATAGATGC-3’ | Mutagenesis |
| CGFYsense | A3G,  A3G-CT | 5'-TCCACATAAACACTGTGGCTTCTACGGCCGCCATGCAGAGCT-3' | Mutagenesis |
| CGFYsense | A3G,  A3G-CT | 5'-AGCTCTGCATGGCGGCCGTAGAAGCCACAGTGTTTATGTGGA-3' | Mutagenesis |
| KNLLCGFYsense | A3GCGFY  A3G-CTCGFY | 5'-CAACCAGGCTAAAAATCTACTCTGTGGCTTCTACGGCCGCCAT-3' | Mutagenesis |
| KNLLCGFYas | A3GCGFY  A3G-CTCGFY | 5'-ATGGCGGCCGTAGAAGCCACAGAGTAGATTTTTAGCCTGGTTG-3' | Mutagenesis |
| YDPsense | A3G,  A3G-CT | 5'-CCGCATCTATGATTATGACCCAAGATGTCAGGAGGGGCT-3' | Mutagenesis |
| YDPas | A3G,  A3G-CT | 5'-AGCCCCTCCTGACATCTTGGGTCATAATCATAGATGCGG-3' | Mutagenesis |
| YDPLYKsense | A3GYDP  A3G-CTYDP | 5'-ATCTATGATTATGACCCCCTATATAAGGAGGGGCTGCGCAC-3' | Mutagenesis |
| YDPLYKas | A3GYDP  A3G-CTYDP | 5'-GTGCGCAGCCCCTCCTTATATAGGGGGTCATAATCATAGAT-3' | Mutagenesis |
| T_28_-TCA-T_29_ | N/A | 5’TTTTTTTTTTTTTTTTTTTTTTTTTTTT**TCA**TTTTTTTTTTTTTTTTTTTTTTTTTTTTT-3’ | D.A. |
| T_28_-CCCG-T_28_ | N/A | 5’TTTTTTTTTTTTTTTTTTTTTTTTTTTT**CCCG**TTTTTTTTTTTTTTTTTTTTTTTTTTTT-3’ | D.A. |
| CCN100 | N/A | 5’TTTTTTTTTTTTTT**CCA**TTTTTTT**CCT**TTTTTTTTT**CCG**TTTTTTTTTTTTTT**CCC**TTTTTTTTTTTTTTTTTTTTTTTTTTTTTTTTTTTTTTTT-3’ | D.A. |
| GCN100 | N/A | 5’TTTTTTTTTTTTTT**GCA**TTTTTTT**GCT**TTTTTTTTT**GCG**TTTTTTTTTTTTTT**GCC**TTTTTTTTTTTTTTTTTTTTTTTTTTTTTTTTTTTTTTTT-3’ | D.A |
| TCN100 | N/A | 5’TTTTTTTTTTTTTT**TCA**TTTTTTT**TCT**TTTTTTTTT**TCG**TTTTTTTTTTTTTT**TCC**TTTTTTTTTTTTTTTTTTTTTTTTTTTTTTTTTTTTTTTT-3’ | D.A. |
| ACN100 | N/A | 5’TTTTTTTTTTTTTT**ACA**TTTTTTT**ACT**TTTTTTTTT**ACG**TTTTTTTTTTTTTT**ACC**TTTTTTTTTTTTTTTTTTTTTTTTTTTTTTTTTTTTTTTT-3’ | D.A. |
| FAM-CCCA-TAMRA | N/A | 6FAM–5’-TTATTATT**CCC**ATTTGATT-3’–TAMRA | FRET |
